# Supplementary material for: Stakeholders’ Perceptions of Biosecurity Implementation in Italian Poultry Farms
Source: Animals (Basel). 2023 Oct 18;13(20):3246. doi: 10.3390/ani13203246 (PMC10603624; doi:10.3390/ani13203246)
Supplement: Supplementary file 1 [file animals-13-03246-s001.zip › Figure S2.pptx]

## Slide 1
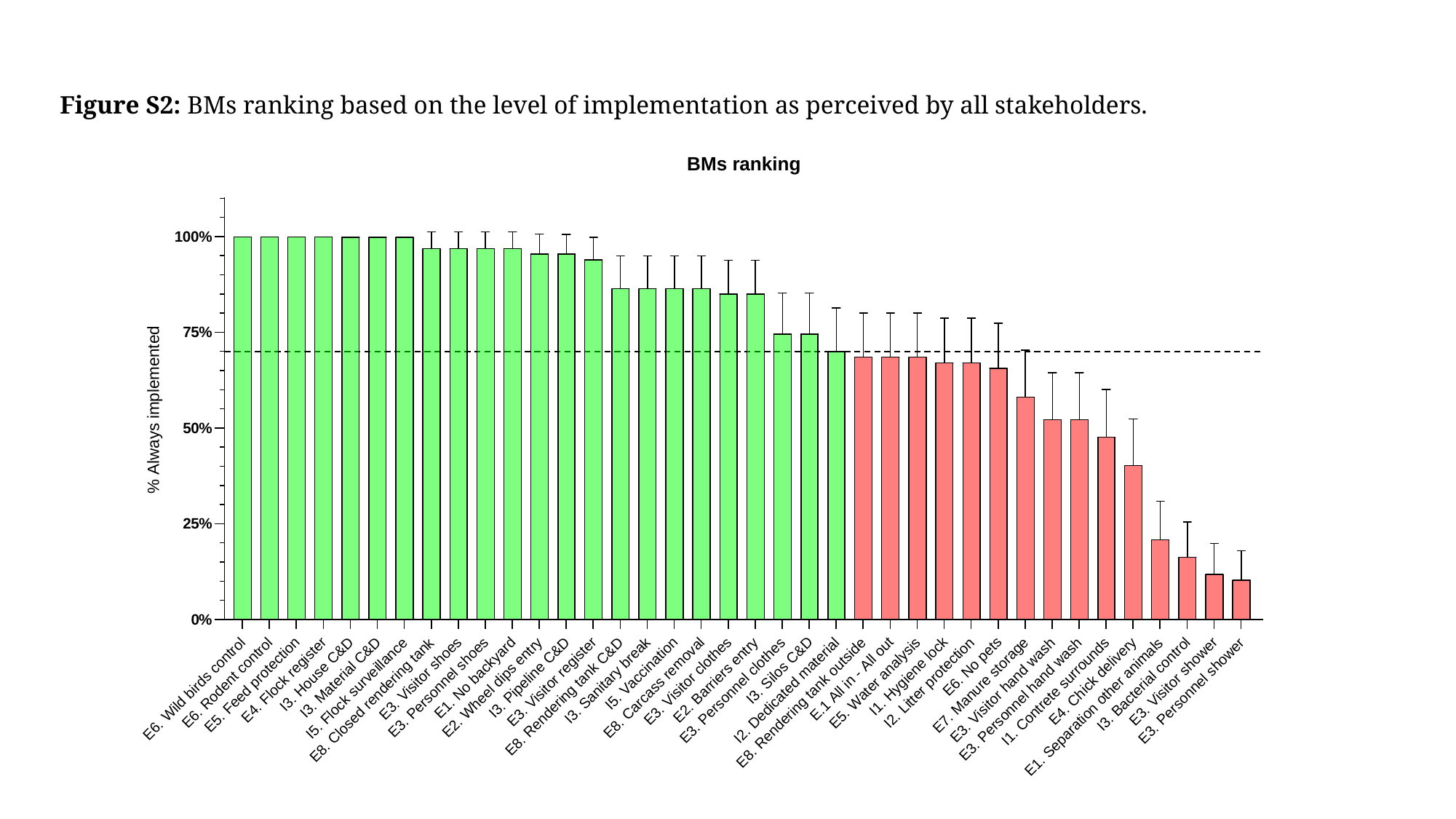

Figure S2: BMs ranking based on the level of implementation as perceived by all stakeholders.
