# Supplementary material for: Stakeholders’ Perceptions of Biosecurity Implementation in Italian Poultry Farms
Source: Animals (Basel). 2023 Oct 18;13(20):3246. doi: 10.3390/ani13203246 (PMC10603624; doi:10.3390/ani13203246)
Supplement: Supplementary file 1 [file animals-13-03246-s001.zip › Table S1_Farmer questionnaire.pdf]

Questionnaire FARMER – NETPOULSAFE (Final - 05/05/2021)

**Objectives and structure of the questionnaire**
**Objectives of the interview:**<br>**-** collect the opinion of the farmers on the implementation of the **biosecurity measures** on their farm, the reasons explaining the difficulties to implement them and their opinion on the efficiency.<br>- collect data on the **supporting measures** that have already helped the farmers to improve the biosecurity on their farm (successful measures) and that the farmers would need (required measures).
**Structure of the questionnaire:**<br><br>**Part 1: Implementation of the Biosecurity Measures on the farm and the reasons of difficulties**<br><br>**Section A:** Similar Biosecurity items - **All the poultry (12 items)**<br>**Section B:** Supplementary Biosecurity item - **Free range (1 item)**<br>**Section C:** Supplementary Biosecurity item - **Breeder (1 item)**<br>**Section D:** Supplementary Biosecurity item - **Layer (1 item)**<br>**Section E:** Other Biosecurity measures not always implemented on the farm - **All the poultry**<br><br>**Part 2: Use of the Supporting Measures by the farmers to improve biosecurity on their farm**<br><br>**Section F**: Successful and Required Supporting measures - **All the poultry (9 items)**<br><br>*(**Item E:** External biosecurity measures, **Item I:** Internal biosecurity measures**, Item S:** Supporting biosecurity measures)*

**Interviewer : Network Facilitator**	
**Country: □ BE Belgium □ ES Spain □ FR France □ HU Hungary □ IT Italy □ NL Netherlands □ PL Poland**	Date of the interview: ………………………………..

**Farmer questioned**
**N° Farmer (1 to X): …………**<br><br>Gender □ man □ woman Age category □ <35 □ 35-55 □ >55

**The Poultry production questioned (1 poultry production)**	
**□ Enclosed Broilers □ Enclosed Turkeys □ Enclosed ducks □ Enclosed layers (enriched cages) □ Enclosed layers (litter system) □ Free range Broilers □ Free range Ducks □ Free range layers □ Breeders □ Other ………………………………………….**<br><br>Details: ……….	
Average capacity of the production on the farm	How many birds? ……………………………<br>How many poultry houses? …………………
Farming system	□ Independent for direct sales<br>□ Under contract with a company for product sales<br>□ Other ………………………………………….
Type of production	□ Standard<br>□ Organic<br>□ Label<br>□ Other ……………………………………………

Section A: Similar Biosecurity items - All the poultry

**Item E1 - Animal production on the site**	**Do you implement these measures on your farm?**	**For a measure not always implemented on your farm**	
		**Could you explain the reasons?**	**Do you think that this measure would be an efficient biosecurity measure for your farm and why?**
"all-in/all-out" poultry production on the site	□ Always<br>□ Sometimes<br>□ Never<br>□ Other frequency…………………………………<br>……………………………………………………….<br>□ Unknown<br>□ Not concerned<br><br>Details: ……………………………………………<br>……………………………………………….........	□ Too expensive<br>□ Take too much time<br>□ Not enough trained<br>□ Not enough advice<br>□ No knowing advantages<br>□ Not useful<br>□ Not adapted to the farm<br>□ Other reason ……………………………………….<br>…………………………………………………………..<br>□ Unknown<br><br>Details: ………………………………………………<br>…………………………………………………………	□ Yes<br>□ Moderately<br>□ No<br>□ Other opinion……………………………………<br>…………………………………………………………<br>□ Unknown<br><br>Why? …………………………………………………<br>…………………………………………………………
No backyard on the site	□ Always<br>□ Sometimes<br>□ Never<br>□ Other frequency…………………………………<br>……………………………………………………….<br>□ Unknown<br>□ Not concerned<br><br>Details: ……………………………………………<br><br>……………………………………………….........	□ Too expensive<br>□ Take too much time<br>□ Not enough trained<br>□ Not enough advice<br>□ No knowing advantages<br>□ Not useful<br>□ Not adapted to the farm<br>□ Other reason ……………………………………….<br>…………………………………………………………..<br>□ Unknown<br><br>Details: ………………………………………………<br>…………………………………………………………	□ Yes<br>□ Moderately<br>□ No<br>□ Other opinion…………………………………….<br>………………………………………………………..<br>□ Unknown<br><br>Why? ………………………………………………...<br>………………………………………………………..
If other animal productions on the site (cattle, pigs) sanitary barriers with poultry (personal, material …)	□ Always<br>□ Sometimes<br>□ Never<br>□ Other frequency…………………………………<br>……………………………………………………….<br>□ Unknown<br>□ Not concerned<br><br>Details: ……………………………………………<br>……………………………………………….........	□ Too expensive<br>□ Take too much time<br>□ Not enough trained<br>□ Not enough advice<br>□ No knowing advantages<br>□ Not useful<br>□ Not adapted to the farm<br>□ Other reason ……………………………………….<br>…………………………………………………………..<br>□ Unknown<br><br>Details: ………………………………………………<br>…………………………………………………………	□ Yes<br>□ Moderately<br>□ No<br>□ Other opinion…………………………………….<br>………………………………………………………..<br>□ Unknown<br><br>Why? …………………………………………………<br>………………………………………………………..

**Item E2 - Structure and circulation on the site**	**Do you implement these measures on your farm?**	**For a measure not always implemented on your farm**	
		**Could you explain the reasons?**	**Do you think that this measure would be an efficient biosecurity measure for your farm and why?**
Delimitation with a barrier or closure of a professional secured area with only necessary vehicles to the poultry house (feed, chicks, poultry or eggs transport vehicles)	☐ Always<br>☐ Sometimes<br>☐ Never<br>☐ Other frequency……………………………<br>……………………………………………..<br>☐ Unknown<br>☐ Not concerned<br><br>Details: ……………………………………<br>……………………………………….……	☐ Too expensive<br>☐ Take too much time<br>☐ Not enough trained<br>☐ Not enough advice<br>☐ No knowing advantages<br>☐ Not useful<br>☐ Not adapted to the farm<br>☐ Other reason ………………………………..<br>……………………………………………..<br>☐ Unknown<br><br>Details: ………………………………………<br>…………………………………………	☐ Yes<br>☐ Moderately<br>☐ No<br>☐ Other opinion……………………………<br>……………………………………………<br>☐ Unknown<br><br>Why? ………………………………………<br>……………………………………………
Wheel dips for disinfection of the vehicles or pulverization before entering on the site	☐ Always<br>☐ Sometimes<br>☐ Never<br>☐ Other frequency……………………………<br>……………………………………………..<br>☐ Unknown<br>☐ Not concerned<br><br>Details: ……………………………………<br>……………………………………….……	☐ Too expensive<br>☐ Take too much time<br>☐ Not enough trained<br>☐ Not enough advice<br>☐ No knowing advantages<br>☐ Not useful<br>☐ Not adapted to the farm<br>☐ Other reason ………………………………..<br>……………………………………………..<br>☐ Unknown<br><br>Details: ………………………………………<br>…………………………………………	☐ Yes<br>☐ Moderately<br>☐ No<br>☐ Other opinion…………………………….<br>……………………………………………..<br>☐ Unknown<br><br>Why? ……………………………………...<br>……………………………………………..
**Item E3 - Personnel, visitors or teams**	**Do you implement these measures on your farm?**	**For a measure not always implemented on your farm**	
		**Could you explain the reasons?**	**Do you think that this measure would be an efficient biosecurity measure for your farm and why?**
**Personnel**			
Specific clothes before entering in the house	☐ Always<br>☐ Sometimes<br>☐ Never<br>☐ Other frequency……………………………<br>……………………………………………..<br>☐ Unknown<br>☐ Not concerned<br><br>Details: ……………………………………<br>……………………………………….……	☐ Too expensive<br>☐ Take too much time<br>☐ Not enough trained<br>☐ Not enough advice<br>☐ No knowing advantages<br>☐ Not useful<br>☐ Not adapted to the farm<br>☐ Other reason ………………………………..<br>……………………………………………..<br>☐ Unknown<br>Details: ………………………………………	☐ Yes<br>☐ Moderately<br>☐ No<br>☐ Other opinion……………………………<br>……………………………………………<br>☐ Unknown<br><br>Why? ………………………………………<br>……………………………………………

Specific shoes before entering in the house	☐ Always<br>☐ Sometimes<br>☐ Never<br>☐ Other frequency…………………………………<br>……………………………………………………..<br>☐ Unknown<br>☐ Not concerned<br><br>Details: ……………………………………………<br><br>………………………………………………..........	☐ Too expensive<br>☐ Take too much time<br>☐ Not enough trained<br>☐ Not enough advice<br>☐ No knowing advantages<br>☐ Not useful<br>☐ Not adapted to the farm<br>☐ Other reason ………………………………………..<br>……………………………………………………….<br>☐ Unknown<br>Details: ………………………………………………	☐ Yes<br>☐ Moderately<br>☐ No<br>☐ Other opinion…………………………………<br>……………………………………………………..<br>☐ Unknown<br><br>Why? ……………………………………………...<br>…………………………………………………….
Washing of the hands before entering in the house	☐ Always<br>☐ Sometimes<br>☐ Never<br>☐ Other frequency…………………………………<br>……………………………………………………..<br>☐ Unknown<br>☐ Not concerned<br><br>Details: ……………………………………………<br><br>………………………………………………..........	☐ Too expensive<br>☐ Take too much time<br>☐ Not enough trained<br>☐ Not enough advice<br>☐ No knowing advantages<br>☐ Not useful<br>☐ Not adapted to the farm<br>☐ Other reason ………………………………………..<br>……………………………………………………….<br>☐ Unknown<br><br>Details: ………………………………………………	☐ Yes<br>☐ Moderately<br>☐ No<br>☐ Other opinion……………………………………<br>………………………………………………………<br>☐ Unknown<br><br>Why? ………………………………………………<br>……………………………………………………….
Showering before entering in the house	☐ Always<br>☐ Sometimes<br>☐ Never<br>☐ Other frequency…………………………………<br>……………………………………………………..<br>☐ Unknown<br>☐ Not concerned<br><br>Details: ……………………………………………<br><br>………………………………………………..........	☐ Too expensive<br>☐ Take too much time<br>☐ Not enough trained<br>☐ Not enough advice<br>☐ No knowing advantages<br>☐ Not useful<br>☐ Not adapted to the farm<br>☐ Other reason ………………………………………..<br>……………………………………………………….<br>☐ Unknown<br>Details: ………………………………………………	☐ Yes<br>☐ Moderately<br>☐ No<br>☐ Other opinion……………………………………<br>………………………………………………………<br>☐ Unknown<br><br>Why? ………………………………………………<br>……………………………………………………….
**Visitors or teams**			
Register for visitors and teams	☐ Always<br>☐ Sometimes<br>☐ Never<br>☐ Other frequency…………………………………<br>……………………………………………………..<br>☐ Unknown<br>☐ Not concerned<br><br>Details: ……………………………………………<br><br>………………………………………………..........	☐ Too expensive<br>☐ Take too much time<br>☐ Not enough trained<br>☐ Not enough advice<br>☐ No knowing advantages<br>☐ Not useful<br>☐ Not adapted to the farm<br>☐ Other reason ………………………………………..<br>……………………………………………………….<br>☐ Unknown<br>Details: ………………………………………………	☐ Yes<br>☐ Moderately<br>☐ No<br>☐ Other opinion……………………………………<br>………………………………………………………<br>☐ Unknown<br><br>Why? ………………………………………………<br>……………………………………………………….

Specific clothes before entering in the house	☐ Always<br>☐ Sometimes<br>☐ Never<br>☐ Other frequency…………………………………<br>………………………………………………………..<br>☐ Unknown<br>☐ Not concerned<br><br>Details: ……………………………………………<br><br>………………………………………………..........	☐ Too expensive<br>☐ Take too much time<br>☐ Not enough trained<br>☐ Not enough advice<br>☐ No knowing advantages<br>☐ Not useful<br>☐ Not adapted to the farm<br>☐ Other reason ………………………………………..<br>…………………………………………………………..<br>☐ Unknown<br>Details: …………………………………………………	☐ Yes<br>☐ Moderately<br>☐ No<br>☐ Other opinion…………………………………….<br>………………………………………………………..<br>☐ Unknown<br><br>Why? ………………………………………………...<br>………………………………………………………..
Specific shoes before entering in the house	☐ Always<br>☐ Sometimes<br>☐ Never<br>☐ Other frequency…………………………………<br>………………………………………………………..<br>☐ Unknown<br>☐ Not concerned<br><br>Details: ……………………………………………<br><br>………………………………………………..........	☐ Too expensive<br>☐ Take too much time<br>☐ Not enough trained<br>☐ Not enough advice<br>☐ No knowing advantages<br>☐ Not useful<br>☐ Not adapted to the farm<br>☐ Other reason ………………………………………..<br>…………………………………………………………..<br>☐ Unknown<br>Details: …………………………………………………	☐ Yes<br>☐ Moderately<br>☐ No<br>☐ Other opinion……………………………………..<br>…………………………………………………………<br>☐ Unknown<br><br>Why? …………………………………………………<br>…………………………………………………………
Washing of the hands before entering in the house	☐ Always<br>☐ Sometimes<br>☐ Never<br>☐ Other frequency…………………………………<br>………………………………………………………..<br>☐ Unknown<br>☐ Not concerned<br><br>Details: ……………………………………………<br><br>………………………………………………..........	☐ Too expensive<br>☐ Take too much time<br>☐ Not enough trained<br>☐ Not enough advice<br>☐ No knowing advantages<br>☐ Not useful<br>☐ Not adapted to the farm<br>☐ Other reason ………………………………………..<br>…………………………………………………………..<br>☐ Unknown<br>Details: …………………………………………………	☐ Yes<br>☐ Moderately<br>☐ No<br>☐ Other opinion……………………………………..<br>…………………………………………………………<br>☐ Unknown<br><br>Why? …………………………………………………<br>…………………………………………………………
Showering before entering in the house	☐ Always<br>☐ Sometimes<br>☐ Never<br>☐ Other frequency…………………………………<br>………………………………………………………..<br>☐ Unknown<br>☐ Not concerned<br><br>Details: ……………………………………………<br><br>………………………………………………..........	☐ Too expensive<br>☐ Take too much time<br>☐ Not enough trained<br>☐ Not enough advice<br>☐ No knowing advantages<br>☐ Not useful<br>☐ Not adapted to the farm<br>☐ Other reason ………………………………………..<br>…………………………………………………………..<br>☐ Unknown<br>Details: ……………………………………………….<br><br>………………………………………………………….	☐ Yes<br>☐ Moderately<br>☐ No<br>☐ Other opinion……………………………………..<br>…………………………………………………………<br>☐ Unknown<br><br>Why? …………………………………………………<br>…………………………………………………………

Item E4 - The poultry at the arrival	Do you implement these measures on your farm?	For a measure not always implemented on your farm	
		Could you explain the reasons?	Do you think that this measure would be an efficient biosecurity measure for your farm and why?
Register for the flock (origin, number of poultry, …)	Always<br>Sometimes<br>Never<br>Other frequency…………………………………<br>……………………………………………………..<br>Unknown<br>Not concerned<br><br>Details: ……………………………………………<br><br>………………………………………………….......	Too expensive<br>Take too much time<br>Not enough trained<br>Not enough advice<br>No knowing advantages<br>Not useful<br>Not adapted to the farm<br>Other reason ………………………………………..<br>…………………………………………………………..<br>Unknown<br>Details: ……………………………………………….<br><br>………………………………………………………….	Yes<br>Moderately<br>No<br>Other opinion……………………………………<br>…………………………………………………………<br>Unknown<br><br>Why? ………………………………………………<br>………………………………………………………….
If the chicks deliverer enters in the house: specific clothes and shoes	Always<br>Sometimes<br>Never<br>Other frequency…………………………………<br>……………………………………………………..<br>Unknown<br>Not concerned<br><br>Details: ……………………………………………<br><br>………………………………………………….......	Too expensive<br>Take too much time<br>Not enough trained<br>Not enough advice<br>No knowing advantages<br>Not useful<br>Not adapted to the farm<br>Other reason ………………………………………..<br>…………………………………………………………..<br>Unknown<br>Details: ……………………………………………….<br><br>………………………………………………………….	Yes<br>Moderately<br>No<br>Other opinion…………………………………….<br>………………………………………………………..<br>Unknown<br><br>Why? ………………………………………………...<br>………………………………………………………..
**Item E5 - Feed and drinking water of the poultry**	**Do you implement these measures on your farm?**	**For a measure not always implemented on your farm**	
		**Could you explain the reasons?**	**Do you think that this measure would be an efficient biosecurity measure for your farm and why?**
Feed storage protection	Always<br>Sometimes<br>Never<br>Other frequency…………………………………<br>……………………………………………………..<br>Unknown<br>Not concerned<br><br>Details: ……………………………………………<br><br>………………………………………………….......	Too expensive<br>Take too much time<br>Not enough trained<br>Not enough advice<br>No knowing advantages<br>Not useful<br>Not adapted to the farm<br>Other reason ………………………………………..<br>…………………………………………………………..<br>Unknown<br>Details: ……………………………………………….<br><br>………………………………………………………….	Yes<br>Moderately<br>No<br>Other opinion……………………………………<br>…………………………………………………………<br>Unknown<br><br>Why? ………………………………………………<br>………………………………………………………….

Drinking water analysis end line each year	☐ Always<br>☐ Sometimes<br>☐ Never<br>☐ Other frequency…………………………………<br>……………………………………………………..<br>☐ Unknown<br>☐ Not concerned<br><br>Details: ……………………………………………<br><br>………………………………………………..........	☐ Too expensive<br>☐ Take too much time<br>☐ Not enough trained<br>☐ Not enough advice<br>☐ No knowing advantages<br>☐ Not useful<br>☐ Not adapted to the farm<br>☐ Other reason ………………………………………..<br>…………………………………………………………..<br>☐ Unknown<br><br>Details: ………………………………………………<br><br>…………………………………………………………	☐ Yes<br>☐ Moderately<br>☐ No<br>☐ Other opinion…………………………………….<br>…………………………………………………………..<br>☐ Unknown<br><br>Why? ………………………………………………...<br>…………………………………………………………..
**Item E6 - Biological vectors control**	**Do you implement these measures on your farm?**	**For a measure not always implemented on your farm**<br>**Could you explain the reasons?**	**Do you think that this measure would be an efficient biosecurity measure for your farm and why?**
Rodents control (deratting or other measures)	☐ Always<br>☐ Sometimes<br>☐ Never<br>☐ Other frequency…………………………………<br>……………………………………………………..<br>☐ Unknown<br>☐ Not concerned<br><br>Details: ……………………………………………<br><br>………………………………………………..........	☐ Too expensive<br>☐ Take too much time<br>☐ Not enough trained<br>☐ Not enough advice<br>☐ No knowing advantages<br>☐ Not useful<br>☐ Not adapted to the farm<br>☐ Other reason ………………………………………..<br>…………………………………………………………..<br>☐ Unknown<br><br>Details: ……………………………………………….<br><br>………………………………………………………….	☐ Yes<br>☐ Moderately<br>☐ No<br>☐ Other opinion………………………………………<br>…………………………………………………………<br>☐ Unknown<br><br>Why? …………………………………………………<br>………………………………………………………….
Wild birds control (protection of the ventilation circuit or other measures)	☐ Always<br>☐ Sometimes<br>☐ Never<br>☐ Other frequency…………………………………<br>……………………………………………………..<br>☐ Unknown<br>☐ Not concerned<br><br>Details: ……………………………………………<br><br>………………………………………………..........	☐ Too expensive<br>☐ Take too much time<br>☐ Not enough trained<br>☐ Not enough advice<br>☐ No knowing advantages<br>☐ Not useful<br>☐ Not adapted to the farm<br>☐ Other reason ………………………………………..<br>…………………………………………………………..<br>☐ Unknown<br><br>Details: ………………………………………………<br><br>…………………………………………………………	☐ Yes<br>☐ Moderately<br>☐ No<br>☐ Other opinion…………………………………….<br>…………………………………………………………..<br>☐ Unknown<br><br>Why? ………………………………………………...<br>…………………………………………………………..

No domestic animals on the site (pets, dogs or cats)	☐ Always<br>☐ Sometimes<br>☐ Never<br>☐ Other frequency…………………………………<br>……………………………………………………….<br>☐ Unknown<br>☐ Not concerned<br><br>Details: ……………………………………………<br><br>………………………………………………..........	☐ Too expensive<br>☐ Take too much time<br>☐ Not enough trained<br>☐ Not enough advice<br>☐ No knowing advantages<br>☐ Not useful<br>☐ Not adapted to the farm<br>☐ Other reason ………………………………………..<br>…………………………………………………………..<br>☐ Unknown<br>Details: ………………………………………………<br><br>…………………………………………………………	☐ Yes<br>☐ Moderately<br>☐ No<br>☐ Other opinion……………………………………<br>…………………………………………………………<br>☐ Unknown<br><br>Why? ………………………………………………<br>………………………………………………………….
**Item E7 - Management of the poultry manure**	**Do you implement these measures on your farm?**	**For a measure not always implemented on your farm**	
		**Could you explain the reasons?**	**Do you think that this measure would be an efficient biosecurity measure for your farm and why?**
Manure stored in a specific isolated area outside of the secured professional area (or if no secured area : away from the house)	☐ Always<br>☐ Sometimes<br>☐ Never<br>☐ Other frequency…………………………………<br>……………………………………………………….<br>☐ Unknown<br>☐ Not concerned<br><br>Details: ……………………………………………<br><br>………………………………………………..........	☐ Too expensive<br>☐ Take too much time<br>☐ Not enough trained<br>☐ Not enough advice<br>☐ No knowing advantages<br>☐ Not useful<br>☐ Not adapted to the farm<br>☐ Other reason ………………………………………..<br>…………………………………………………………..<br>☐ Unknown<br>Details: ………………………………………………<br><br>…………………………………………………………	☐ Yes<br>☐ Moderately<br>☐ No<br>☐ Other opinion……………………………………<br>…………………………………………………………<br>☐ Unknown<br><br>Why? ………………………………………………<br>………………………………………………………….
**Item E8 - Management of dead animals**	**Do you implement these measures on your farm?**	**For a measure not always implemented on your farm**	
		**Could you explain the reasons?**	**Do you think that this measure would be an efficient biosecurity measure for your farm and why?**
Removal of the carcasses at least twice a day	☐ Always<br>☐ Sometimes<br>☐ Never<br>☐ Other frequency…………………………………<br>……………………………………………………….<br>☐ Unknown<br>☐ Not concerned<br><br>Details: ……………………………………………<br><br>………………………………………………..........	☐ Too expensive<br>☐ Take too much time<br>☐ Not enough trained<br>☐ Not enough advice<br>☐ No knowing advantages<br>☐ Not useful<br>☐ Not adapted to the farm<br>☐ Other reason ………………………………………..<br>…………………………………………………………..<br>☐ Unknown<br><br>Details: ………………………………………………<br><br>…………………………………………………………	☐ Yes<br>☐ Moderately<br>☐ No<br>☐ Other opinion……………………………………<br>…………………………………………………………<br>☐ Unknown<br><br>Why? ………………………………………………<br>………………………………………………………….

Presence of a closed and protected rendering tank	☐ Always<br>☐ Sometimes<br>☐ Never<br>☐ Other frequency…………………………………<br>………………………………………………………..<br>☐ Unknown<br>☐ Not concerned<br><br>Details: ……………………………………………<br><br>………………………………………………..........	☐ Too expensive<br>☐ Take too much time<br>☐ Not enough trained<br>☐ Not enough advice<br>☐ No knowing advantages<br>☐ Not useful<br>☐ Not adapted to the farm<br>☐ Other reason ……………………………………..<br>………………………………………………………..<br>☐ Unknown<br><br>Details: ………………………………………………<br><br>…………………………………………………………	☐ Yes<br>☐ Moderately<br>☐ No<br>☐ Other opinion…………………………………….<br>………………………………………………………..<br>☐ Unknown<br><br>Why? ………………………………………………...<br>………………………………………………………..
Rendering tank located outside of the secured area (or if no secured area : away from the house) allowing the passage of the truck away from the house	☐ Always<br>☐ Sometimes<br>☐ Never<br>☐ Other frequency…………………………………<br>………………………………………………………..<br>☐ Unknown<br>☐ Not concerned<br><br>Details: ……………………………………………<br><br>………………………………………………..........	☐ Too expensive<br>☐ Take too much time<br>☐ Not enough trained<br>☐ Not enough advice<br>☐ No knowing advantages<br>☐ Not useful<br>☐ Not adapted to the farm<br>☐ Other reason ……………………………………..<br>………………………………………………………..<br>☐ Unknown<br><br>Details: ………………………………………………<br><br>………………………………………………………….	☐ Yes<br>☐ Moderately<br>☐ No<br>☐ Other opinion…………………………………….<br>…………………………………………………………<br>☐ Unknown<br><br>Why? ……………………………………………….<br>………………………………………………………….
Cleaning and disinfection of the rendering tank after each collection	☐ Always<br>☐ Sometimes<br>☐ Never<br>☐ Other frequency…………………………………<br>………………………………………………………..<br>☐ Unknown<br>☐ Not concerned<br><br>Details: ……………………………………………<br><br>………………………………………………..........	☐ Too expensive<br>☐ Take too much time<br>☐ Not enough trained<br>☐ Not enough advice<br>☐ No knowing advantages<br>☐ Not useful<br>☐ Not adapted to the farm<br>☐ Other reason ……………………………………..<br>………………………………………………………..<br>☐ Unknown<br><br>Details: ………………………………………………<br><br>………………………………………………………….	☐ Yes<br>☐ Moderately<br>☐ No<br>☐ Other opinion…………………………………….<br>…………………………………………………………<br>☐ Unknown<br><br>Why? ……………………………………………….<br>………………………………………………………….

Item I1 - Structure and circulation in the poultry house	Do you implement these measures on your farm?	For a measure not always implemented on your farm<br>Could you explain the reasons?	Do you think that this measure would be an efficient biosecurity measure for your farm and why?
Concrete surrounds around the house	☐ Always<br>☐ Sometimes<br>☐ Never<br>☐ Other frequency…………………………………<br>………………………………………………………..<br>☐ Unknown<br>☐ Not concerned<br><br>Details: ……………………………………………<br><br>…………………………………………………........	☐ Too expensive<br>☐ Take too much time<br>☐ Not enough trained<br>☐ Not enough advice<br>☐ No knowing advantages<br>☐ Not useful<br>☐ Not adapted to the farm<br>☐ Other reason ………………………………………..<br>…………………………………………………………..<br>☐ Unknown<br><br>Details: ……………………………………………….<br><br>…………………………………………………………	☐ Yes<br>☐ Moderately<br>☐ No<br>☐ Other opinion…………………………………….<br>………………………………………………………..<br>☐ Unknown<br><br>Why? ………………………………………………...<br>………………………………………………………..
Hygiene lock with 2 separated zones (clean and dirty area)	☐ Always<br>☐ Sometimes<br>☐ Never<br>☐ Other frequency…………………………………<br>………………………………………………………..<br>☐ Unknown<br>☐ Not concerned<br><br>Details: ……………………………………………<br><br>…………………………………………………........	☐ Too expensive<br>☐ Take too much time<br>☐ Not enough trained<br>☐ Not enough advice<br>☐ No knowing advantages<br>☐ Not useful<br>☐ Not adapted to the farm<br>☐ Other reason ………………………………………..<br>…………………………………………………………..<br>☐ Unknown<br><br>Details: ……………………………………………….<br><br>………………………………………………………….	☐ Yes<br>☐ Moderately<br>☐ No<br>☐ Other opinion……………………………………<br>…………………………………………………………<br>☐ Unknown<br><br>Why? …………………………………………………<br>………………………………………………………….

**Item I2 - Management of the material or litter in the poultry house**	**Do you implement these measures on your farm?**	**For a measure not always implemented on your farm**	
		**Could you explain the reasons?**	**Do you think that this measure would be an efficient biosecurity measure for your farm and why?**
Recognizable separate material only for the poultry house	☐ Always<br>☐ Sometimes<br>☐ Never<br>☐ Other frequency…………………………………<br>………………………………………………………..<br>☐ Unknown<br>☐ Not concerned<br><br>Details: ……………………………………………<br><br>………………………………………………........	☐ Too expensive<br>☐ Take too much time<br>☐ Not enough trained<br>☐ Not enough advice<br>☐ No knowing advantages<br>☐ Not useful<br>☐ Not adapted to the farm<br>☐ Other reason ………………………………………..<br>………………………………………………………..<br>☐ Unknown<br>Details: ………………………………………………<br><br>…………………………………………………………	☐ Yes<br>☐ Moderately<br>☐ No<br>☐ Other opinion……………………………………<br>…………………………………………………………<br>☐ Unknown<br><br>Why? ………………………………………………<br>………………………………………………………….
Protection of the litter (in a closed shed or other protection, from birds or vermin …)	☐ Always<br>☐ Sometimes<br>☐ Never<br>☐ Other frequency…………………………………<br>………………………………………………………..<br>☐ Unknown<br>☐ Not concerned<br><br>Details: ……………………………………………<br><br>………………………………………………........	☐ Too expensive<br>☐ Take too much time<br>☐ Not enough trained<br>☐ Not enough advice<br>☐ No knowing advantages<br>☐ Not useful<br>☐ Not adapted to the farm<br>☐ Other reason ………………………………………..<br>………………………………………………………..<br>☐ Unknown<br>Details: ………………………………………………<br><br>…………………………………………………………	☐ Yes<br>☐ Moderately<br>☐ No<br>☐ Other opinion…………………………………….<br>………………………………………………………..<br>☐ Unknown<br><br>Why? ………………………………………………...<br>………………………………………………………..

**Item I3 - Cleaning and disinfection of the house and material**	**Do you implement these measures on your farm?**	**For a measure not always implemented on your farm**	
		**Could you explain the reasons?**	**Do you think that this measure would be an efficient biosecurity measure for your farm and why?**
Cleaning and disinfection of the house between each flock	☐ Always<br>☐ Sometimes<br>☐ Never<br>☐ Other frequency…………………………………<br>………………………………………………………..<br>☐ Unknown<br>☐ Not concerned<br><br>Details: ……………………………………………<br><br>………………………………………………........	☐ Too expensive<br>☐ Take too much time<br>☐ Not enough trained<br>☐ Not enough advice<br>☐ No knowing advantages<br>☐ Not useful<br>☐ Not adapted to the farm<br>☐ Other reason ………………………………………..<br>………………………………………………………..<br>☐ Unknown<br><br>Details: ………………………………………………<br><br>…………………………………………………………	☐ Yes<br>☐ Moderately<br>☐ No<br>☐ Other opinion……………………………………<br>…………………………………………………………<br>☐ Unknown<br><br>Why? ………………………………………………<br>………………………………………………………….

Cleaning and disinfection of the material between each flock (feeders, drinkers, nests, material for the management of eggs, …)	□ Always<br>□ Sometimes<br>□ Never<br>□ Other frequency…………………………………<br>………………………………………………………..<br>□ Unknown<br>□ Not concerned<br><br>Details: ……………………………………………<br><br>………………………………………………….........	□ Too expensive<br>□ Take too much time<br>□ Not enough trained<br>□ Not enough advice<br>□ No knowing advantages<br>□ Not useful<br>□ Not adapted to the farm<br>□ Other reason ………………………………………..<br>…………………………………………………………..<br>□ Unknown<br>Details: ……………………………………………….	□ Yes<br>□ Moderately<br>□ No<br>□ Other opinion…………………………………….<br>………………………………………………………..<br>□ Unknown<br><br>Why? ………………………………………………...<br>………………………………………………………..
Cleaning and disinfection of the drinking water pipeline between each flock	□ Always<br>□ Sometimes<br>□ Never<br>□ Other frequency…………………………………<br>………………………………………………………..<br>□ Unknown<br>□ Not concerned<br><br>Details: ……………………………………………<br><br>………………………………………………….........	□ Too expensive<br>□ Take too much time<br>□ Not enough trained<br>□ Not enough advice<br>□ No knowing advantages<br>□ Not useful<br>□ Not adapted to the farm<br>□ Other reason ………………………………………..<br>…………………………………………………………..<br>□ Unknown<br>Details: ……………………………………………….	□ Yes<br>□ Moderately<br>□ No<br>□ Other opinion……………………………………..<br>…………………………………………………………<br>□ Unknown<br><br>Why? …………………………………………………<br>…………………………………………………………
Cleaning and disinfection of the feed silo between each flock	□ Always<br>□ Sometimes<br>□ Never<br>□ Other frequency…………………………………<br>………………………………………………………..<br>□ Unknown<br>□ Not concerned<br><br>Details: ……………………………………………<br><br>………………………………………………….........	□ Too expensive<br>□ Take too much time<br>□ Not enough trained<br>□ Not enough advice<br>□ No knowing advantages<br>□ Not useful<br>□ Not adapted to the farm<br>□ Other reason ………………………………………..<br>…………………………………………………………..<br>□ Unknown<br>Details: ……………………………………………….	□ Yes<br>□ Moderately<br>□ No<br>□ Other opinion…………………………………….<br>………………………………………………………..<br>□ Unknown<br><br>Why? ………………………………………………...<br>………………………………………………………..
Bacterial autocontrol of the cleaning and disinfection of the house between each flock	□ Always<br>□ Sometimes<br>□ Never<br>□ Other frequency…………………………………<br>………………………………………………………..<br>□ Unknown<br>□ Not concerned<br><br>Details: ……………………………………………<br><br>………………………………………………….........	□ Too expensive<br>□ Take too much time<br>□ Not enough trained<br>□ Not enough advice<br>□ No knowing advantages<br>□ Not useful<br>□ Not adapted to the farm<br>□ Other reason ………………………………………..<br>…………………………………………………………..<br>□ Unknown<br><br>Details: ……………………………………………….<br><br>………………………………………………………….	□ Yes<br>□ Moderately<br>□ No<br>□ Other opinion……………………………………..<br>…………………………………………………………<br>□ Unknown<br><br>Why? …………………………………………………<br>…………………………………………………………

Period of the sanitary break > 15 days between each flock	☐ Always<br>☐ Sometimes<br>☐ Never<br>☐ Other frequency…………………………………<br>……………………………………………………..<br>☐ Unknown<br>☐ Not concerned<br><br>Details: ……………………………………………<br><br>………………………………………………..........	☐ Too expensive<br>☐ Take too much time<br>☐ Not enough trained<br>☐ Not enough advice<br>☐ No knowing advantages<br>☐ Not useful<br>☐ Not adapted to the farm<br>☐ Other reason ………………………………………..<br>…………………………………………………………..<br>☐ Unknown<br><br>Details: ………………………………………………<br><br>…………………………………………………………	☐ Yes<br>☐ Moderately<br>☐ No<br>☐ Other opinion…………………………………….<br>……………………………………………………….<br>☐ Unknown<br><br>Why? ………………………………………………...<br>………………………………………………………..
**Item I5 - Management of the poultry**	**Do you implement these measures on your farm?**	**For a measure not always implemented on your farm**<br>**Could you explain the reasons?**	**Do you think that this measure would be an efficient biosecurity measure for your farm and why?**
Vaccination protocol of each poultry flock	☐ Always<br>☐ Sometimes<br>☐ Never<br>☐ Other frequency…………………………………<br>……………………………………………………..<br>☐ Unknown<br>☐ Not concerned<br><br>Details: ……………………………………………<br><br>………………………………………………..........	☐ Too expensive<br>☐ Take too much time<br>☐ Not enough trained<br>☐ Not enough advice<br>☐ No knowing advantages<br>☐ Not useful<br>☐ Not adapted to the farm<br>☐ Other reason ………………………………………..<br>…………………………………………………………..<br>☐ Unknown<br><br>Details: ……………………………………………….<br><br>………………………………………………………….	☐ Yes<br>☐ Moderately<br>☐ No<br>☐ Other opinion……………………………………….<br>…………………………………………………………<br>☐ Unknown<br><br>Why? …………………………………………………<br>………………………………………………………….
Daily surveillance with clinical alert criteria (water and feed consummation, mortality, eggs production)	☐ Always<br>☐ Sometimes<br>☐ Never<br>☐ Other frequency…………………………………<br>……………………………………………………..<br>☐ Unknown<br>☐ Not concerned<br><br>Details: ……………………………………………<br><br>………………………………………………..........	☐ Too expensive<br>☐ Take too much time<br>☐ Not enough trained<br>☐ Not enough advice<br>☐ No knowing advantages<br>☐ Not useful<br>☐ Not adapted to the farm<br>☐ Other reason ………………………………………..<br>…………………………………………………………..<br>☐ Unknown<br><br>Details: ………………………………………………<br><br>…………………………………………………………	☐ Yes<br>☐ Moderately<br>☐ No<br>☐ Other opinion…………………………………….<br>……………………………………………………….<br>☐ Unknown<br><br>Why? ………………………………………………...<br>………………………………………………………..

Section B. Supplementary Biosecurity Item - Free range

**Item I4 - Management of the free range area**	**Do you implement these measures on your farm?**	**For a measure not always implemented on your farm**<br>**Could you explain the reasons?**	**Do you think that this measure would be an efficient biosecurity measure for your farm and why?**
Free range area in the secured professional area without vehicle passage	☐ Always<br>☐ Sometimes<br>☐ Never<br>☐ Other frequency…………………………………<br>………………………………………………………..<br>☐ Unknown<br>☐ Not concerned<br><br>Details: ……………………………………………<br><br>………………………………………………..........	☐ Too expensive<br>☐ Take too much time<br>☐ Not enough trained<br>☐ Not enough advice<br>☐ No knowing advantages<br>☐ Not useful<br>☐ Not adapted to the farm<br>☐ Other reason ……………………………………..<br>…………………………………………………………..<br>☐ Unknown<br>Details: ………………………………………………<br><br>…………………………………………………………	☐ Yes<br>☐ Moderately<br>☐ No<br>☐ Other opinion……………………………………<br>………………………………………………………<br>☐ Unknown<br><br>Why? …………………………………………………<br>………………………………………………………….
Free range area away from a waterway	☐ Always<br>☐ Sometimes<br>☐ Never<br>☐ Other frequency…………………………………<br>………………………………………………………..<br>☐ Unknown<br>☐ Not concerned<br><br>Details: ……………………………………………<br><br>………………………………………………..........	☐ Too expensive<br>☐ Take too much time<br>☐ Not enough trained<br>☐ Not enough advice<br>☐ No knowing advantages<br>☐ Not useful<br>☐ Not adapted to the farm<br>☐ Other reason ……………………………………..<br>…………………………………………………………..<br>☐ Unknown<br>Details: ……………………………………………….<br><br>…………………………………………………………	☐ Yes<br>☐ Moderately<br>☐ No<br>☐ Other opinion…………………………………….<br>…………………………………………………………..<br>☐ Unknown<br><br>Why? ………………………………………………...<br>………………………………………………………..
Free range area closed	☐ Always<br>☐ Sometimes<br>☐ Never<br>☐ Other frequency…………………………………<br>………………………………………………………..<br>☐ Unknown<br>☐ Not concerned<br><br>Details: ……………………………………………<br><br>………………………………………………..........	☐ Too expensive<br>☐ Take too much time<br>☐ Not enough trained<br>☐ Not enough advice<br>☐ No knowing advantages<br>☐ Not useful<br>☐ Not adapted to the farm<br>☐ Other reason ……………………………………..<br>…………………………………………………………..<br>☐ Unknown<br>Details: ………………………………………………<br><br>…………………………………………………………	☐ Yes<br>☐ Moderately<br>☐ No<br>☐ Other opinion……………………………………<br>………………………………………………………<br>☐ Unknown<br><br>Why? …………………………………………………<br>………………………………………………………….

Concrete exit area to avoid stagnant water in front of the house	□ Always<br>□ Sometimes<br>□ Never<br>□ Other frequency…………………………………<br>…………………………………………………………..<br>□ Unknown<br>□ Not concerned<br><br>Details: ……………………………………………<br><br>…………………………………………………….........	□ Too expensive<br>□ Take too much time<br>□ Not enough trained<br>□ Not enough advice<br>□ No knowing advantages<br>□ Not useful<br>□ Not adapted to the farm<br>□ Other reason ………………………………………..<br>……………………………………………………………..<br>□ Unknown<br>Details: ………………………………………………	□ Yes<br>□ Moderately<br>□ No<br>□ Other opinion……………………………………<br>…………………………………………………………..<br>□ Unknown<br><br>Why? ………………………………………………...<br>…………………………………………………………..
No stagnant water on the free range area	□ Always<br>□ Sometimes<br>□ Never<br>□ Other frequency…………………………………<br>…………………………………………………………..<br>□ Unknown<br>□ Not concerned<br><br>Details: ……………………………………………<br><br>…………………………………………………….........	□ Too expensive<br>□ Take too much time<br>□ Not enough trained<br>□ Not enough advice<br>□ No knowing advantages<br>□ Not useful<br>□ Not adapted to the farm<br>□ Other reason ………………………………………..<br>……………………………………………………………..<br>□ Unknown<br>Details: ………………………………………………	□ Yes<br>□ Moderately<br>□ No<br>□ Other opinion……………………………………<br>…………………………………………………………<br>□ Unknown<br><br>Why? ………………………………………………<br>……………………………………………………………
Protective net on the free range area (with anti-roost system)	□ Always<br>□ Sometimes<br>□ Never<br>□ Other frequency…………………………………<br>…………………………………………………………..<br>□ Unknown<br>□ Not concerned<br><br>Details: ……………………………………………<br><br>…………………………………………………….........	□ Too expensive<br>□ Take too much time<br>□ Not enough trained<br>□ Not enough advice<br>□ No knowing advantages<br>□ Not useful<br>□ Not adapted to the farm<br>□ Other reason ………………………………………..<br>……………………………………………………………..<br>□ Unknown<br>Details: ………………………………………………	□ Yes<br>□ Moderately<br>□ No<br>□ Other opinion……………………………………<br>…………………………………………………………..<br>□ Unknown<br><br>Why? ………………………………………………...<br>…………………………………………………………..
No food and drinking water outside the house	□ Always<br>□ Sometimes<br>□ Never<br>□ Other frequency…………………………………<br>…………………………………………………………..<br>□ Unknown<br>□ Not concerned<br><br>Details: ……………………………………………<br><br>…………………………………………………….........	□ Too expensive<br>□ Take too much time<br>□ Not enough trained<br>□ Not enough advice<br>□ No knowing advantages<br>□ Not useful<br>□ Not adapted to the farm<br>□ Other reason ………………………………………..<br>……………………………………………………………..<br>□ Unknown<br><br>Details: ………………………………………………<br><br>……………………………………………………………	□ Yes<br>□ Moderately<br>□ No<br>□ Other opinion……………………………………<br>…………………………………………………………<br>□ Unknown<br><br>Why? ………………………………………………<br>……………………………………………………………

Maintenance and disinfection of the free range area	☐ Always<br>☐ Sometimes<br>☐ Never<br>☐ Other frequency…………………………………<br>………………………………………………………..<br>☐ Unknown<br>☐ Not concerned<br><br>Details: ……………………………………………<br><br>………………………………………………….........	☐ Too expensive<br>☐ Take too much time<br>☐ Not enough trained<br>☐ Not enough advice<br>☐ No knowing advantages<br>☐ Not useful<br>☐ Not adapted to the farm<br>☐ Other reason ……………………………………….<br>…………………………………………………………..<br>☐ Unknown<br><br>Details: ……………………………………………….<br><br>……………………………………………………………	☐ Yes<br>☐ Moderately<br>☐ No<br>☐ Other opinion…………………………………….<br>………………………………………………………..<br>☐ Unknown<br><br>Why? …………………………………………………...<br>………………………………………………………..
Period of sanitary break of the free range area	☐ Always<br>☐ Sometimes<br>☐ Never<br>☐ Other frequency…………………………………<br>………………………………………………………..<br>☐ Unknown<br>☐ Not concerned<br><br>Details: ……………………………………………<br><br>………………………………………………….........	☐ Too expensive<br>☐ Take too much time<br>☐ Not enough trained<br>☐ Not enough advice<br>☐ No knowing advantages<br>☐ Not useful<br>☐ Not adapted to the farm<br>☐ Other reason ……………………………………….<br>…………………………………………………………..<br>☐ Unknown<br><br>Details: ……………………………………………….<br><br>……………………………………………………………	☐ Yes<br>☐ Moderately<br>☐ No<br>☐ Other opinion…………………………………….<br>………………………………………………………..<br>☐ Unknown<br><br>Why? …………………………………………………...<br>………………………………………………………..

Section C.Supplementary Biosecurity Item - Breeder

**Item I5 - Management of the hatching eggs**	**Do you implement these measures on your farm?**	**For a measure not always implemented on your farm**	
		**Could you explain the reasons?**	**Do you think that this measure would be an efficient biosecurity measure for your farm and why?**
Automatic hatching eggs collection system	☐ Always<br>☐ Sometimes<br>☐ Never<br>☐ Other frequency…………………………………<br>………………………………………………………..<br>☐ Unknown<br>☐ Not concerned<br><br>Details: ……………………………………………<br>……………………………………………..........	☐ Too expensive<br>☐ Take too much time<br>☐ Not enough trained<br>☐ Not enough advice<br>☐ No knowing advantages<br>☐ Not useful<br>☐ Not adapted to the farm<br>☐ Other reason ………………………………………..<br>………………………………………………………..<br>☐ Unknown<br>Details: ………………………………………………<br>…………………………………………………………	☐ Yes<br>☐ Moderately<br>☐ No<br>☐ Other opinion……………………………………<br>………………………………………………………..<br>☐ Unknown<br><br>Why? ………………………………………………...<br>………………………………………………………..
Traceability of the hatching eggs (breeder flock, laying date …)	☐ Always<br>☐ Sometimes<br>☐ Never<br>☐ Other frequency…………………………………<br>………………………………………………………..<br>☐ Unknown<br>☐ Not concerned<br><br>Details: ……………………………………………<br>……………………………………………..........	☐ Too expensive<br>☐ Take too much time<br>☐ Not enough trained<br>☐ Not enough advice<br>☐ No knowing advantages<br>☐ Not useful<br>☐ Not adapted to the farm<br>☐ Other reason ………………………………………..<br>………………………………………………………..<br>☐ Unknown<br><br>Details: ………………………………………………<br>…………………………………………………………	☐ Yes<br>☐ Moderately<br>☐ No<br>☐ Other opinion……………………………………<br>………………………………………………………..<br>☐ Unknown<br><br>Why? ………………………………………………...<br>………………………………………………………..
Hatching eggs disinfection at the farm	☐ Always<br>☐ Sometimes<br>☐ Never<br>☐ Other frequency…………………………………<br>………………………………………………………..<br>☐ Unknown<br>☐ Not concerned<br><br>Details: ……………………………………………<br>……………………………………………..........	☐ Too expensive<br>☐ Take too much time<br>☐ Not enough trained<br>☐ Not enough advice<br>☐ No knowing advantages<br>☐ Not useful<br>☐ Not adapted to the farm<br>☐ Other reason ………………………………………..<br>………………………………………………………..<br>☐ Unknown<br>Details: ………………………………………………<br>…………………………………………………………	☐ Yes<br>☐ Moderately<br>☐ No<br>☐ Other opinion……………………………………<br>………………………………………………………..<br>☐ Unknown<br><br>Why? ………………………………………………...<br>………………………………………………………..

Specific hatching eggs sorting and storage rooms	☐ Always<br>☐ Sometimes<br>☐ Never<br>☐ Other frequency…………………………………<br>………………………………………………………..<br>☐ Unknown<br>☐ Not concerned<br><br>Details: ……………………………………………<br><br>………………………………………………..........	☐ Too expensive<br>☐ Take too much time<br>☐ Not enough trained<br>☐ Not enough advice<br>☐ No knowing advantages<br>☐ Not useful<br>☐ Not adapted to the farm<br>☐ Other reason ………………………………………..<br>…………………………………………………………..<br>☐ Unknown<br>Details: ………………………………………………	☐ Yes<br>☐ Moderately<br>☐ No<br>☐ Other opinion……………………………………<br>………………………………………………………..<br>☐ Unknown<br><br>Why? ………………………………………………...<br>………………………………………………………..
Specific entrance for the collection of the hatching eggs by the driver	☐ Always<br>☐ Sometimes<br>☐ Never<br>☐ Other frequency…………………………………<br>………………………………………………………..<br>☐ Unknown<br>☐ Not concerned<br><br>Details: ……………………………………………<br><br>………………………………………………..........	☐ Too expensive<br>☐ Take too much time<br>☐ Not enough trained<br>☐ Not enough advice<br>☐ No knowing advantages<br>☐ Not useful<br>☐ Not adapted to the farm<br>☐ Other reason ………………………………………..<br>…………………………………………………………..<br>☐ Unknown<br>Details: ………………………………………………	☐ Yes<br>☐ Moderately<br>☐ No<br>☐ Other opinion……………………………………<br>………………………………………………………..<br>☐ Unknown<br><br>Why? ………………………………………………...<br>………………………………………………………..
If the driver enters in the storage rooms for the collection of eggs: specific clothes and shoes	☐ Always<br>☐ Sometimes<br>☐ Never<br>☐ Other frequency…………………………………<br>………………………………………………………..<br>☐ Unknown<br>☐ Not concerned<br><br>Details: ……………………………………………<br><br>………………………………………………..........	☐ Too expensive<br>☐ Take too much time<br>☐ Not enough trained<br>☐ Not enough advice<br>☐ No knowing advantages<br>☐ Not useful<br>☐ Not adapted to the farm<br>☐ Other reason ………………………………………..<br>…………………………………………………………..<br>☐ Unknown<br>Details: ………………………………………………	☐ Yes<br>☐ Moderately<br>☐ No<br>☐ Other opinion……………………………………<br>………………………………………………………..<br>☐ Unknown<br><br>Why? ………………………………………………...<br>………………………………………………………..
Cleaning and disinfection of the storage room after each collection	☐ Always<br>☐ Sometimes<br>☐ Never<br>☐ Other frequency…………………………………<br>………………………………………………………..<br>☐ Unknown<br>☐ Not concerned<br><br>Details: ……………………………………………<br><br>………………………………………………..........	☐ Too expensive<br>☐ Take too much time<br>☐ Not enough trained<br>☐ Not enough advice<br>☐ No knowing advantages<br>☐ Not useful<br>☐ Not adapted to the farm<br>☐ Other reason ………………………………………..<br>…………………………………………………………..<br>☐ Unknown<br><br>Details: ………………………………………………<br><br>…………………………………………………………	☐ Yes<br>☐ Moderately<br>☐ No<br>☐ Other opinion……………………………………<br>………………………………………………………..<br>☐ Unknown<br><br>Why? ………………………………………………...<br>………………………………………………………..

Different personal between the poultry room and the eggs sorting and storage rooms	□ Always<br>□ Sometimes<br>□ Never<br>□ Other frequency…………………………………<br>…………………………………………………………..<br>□ Unknown<br>□ Not concerned<br><br>Details: ……………………………………………<br><br>………………………………………………..........	□ Too expensive<br>□ Take too much time<br>□ Not enough trained<br>□ Not enough advice<br>□ No knowing advantages<br>□ Not useful<br>□ Not adapted to the farm<br>□ Other reason ………………………………………..<br>…………………………………………………………..<br>□ Unknown<br><br>Details: ……………………………………………….<br><br>………………………………………………………….	□ Yes<br>□ Moderately<br>□ No<br>□ Other opinion……………………………………<br>…………………………………………………………..<br>□ Unknown<br><br>Why? ………………………………………………...<br>…………………………………………………………..

Section D.Supplementary Biosecurity Item - Layers

**Item I5 - Management of the laying eggs**	**Do you implement these measures on your farm?**	**For a measure not always implemented on your farm**	
		**Could you explain the reasons?**	**Do you think that this measure would be an efficient biosecurity measure for your farm and why?**
Automatic laying eggs collection system	☐ Always<br>☐ Sometimes<br>☐ Never<br>☐ Other frequency…………………………………<br>………………………………………………………..<br>☐ Unknown<br>☐ Not concerned<br><br>Details: ……………………………………………<br>………………………………………………..........	☐ Too expensive<br>☐ Take too much time<br>☐ Not enough trained<br>☐ Not enough advice<br>☐ No knowing advantages<br>☐ Not useful<br>☐ Not adapted to the farm<br>☐ Other reason ………………………………………..<br>…………………………………………………………..<br>☐ Unknown<br><br>Details: ………………………………………………	☐ Yes<br>☐ Moderately<br>☐ No<br>☐ Other opinion……………………………………<br>………………………………………………………..<br>☐ Unknown<br><br>Why? ………………………………………………...<br>………………………………………………………..
Traceability of the laying eggs (layers flock, laying date …)	☐ Always<br>☐ Sometimes<br>☐ Never<br>☐ Other frequency…………………………………<br>………………………………………………………..<br>☐ Unknown<br>☐ Not concerned<br><br>Details: ……………………………………………<br>………………………………………………..........	☐ Too expensive<br>☐ Take too much time<br>☐ Not enough trained<br>☐ Not enough advice<br>☐ No knowing advantages<br>☐ Not useful<br>☐ Not adapted to the farm<br>☐ Other reason ………………………………………..<br>…………………………………………………………..<br>☐ Unknown<br><br>Details: ………………………………………………<br>…………………………………………………………	☐ Yes<br>☐ Moderately<br>☐ No<br>☐ Other opinion……………………………………<br>………………………………………………………..<br>☐ Unknown<br><br>Why? ………………………………………………...<br>………………………………………………………..
Specific laying eggs sorting and storage rooms	☐ Always<br>☐ Sometimes<br>☐ Never<br>☐ Other frequency…………………………………<br>………………………………………………………..<br>☐ Unknown<br>☐ Not concerned<br><br>Details: ……………………………………………<br>………………………………………………..........	☐ Too expensive<br>☐ Take too much time<br>☐ Not enough trained<br>☐ Not enough advice<br>☐ No knowing advantages<br>☐ Not useful<br>☐ Not adapted to the farm<br>☐ Other reason ………………………………………..<br>…………………………………………………………..<br>☐ Unknown<br><br>Details: ………………………………………………	☐ Yes<br>☐ Moderately<br>☐ No<br>☐ Other opinion……………………………………<br>………………………………………………………..<br>☐ Unknown<br><br>Why? ………………………………………………...<br>………………………………………………………..

Specific entrance for the collection of the eggs by the driver	□ Always<br>□ Sometimes<br>□ Never<br>□ Other frequency………………………………<br>…………………………………………………….<br>□ Unknown<br>□ Not concerned<br><br>Details: ……………………………………………<br><br>……………………………………………........	□ Too expensive<br>□ Take too much time<br>□ Not enough trained<br>□ Not enough advice<br>□ No knowing advantages<br>□ Not useful<br>□ Not adapted to the farm<br>□ Other reason ………………………………………..<br>……………………………………………………………..<br>□ Unknown<br>Details: ………………………………………………<br><br>………………………………………………………	□ Yes<br>□ Moderately<br>□ No<br>□ Other opinion…………………………………<br>……………………………………………………..<br>□ Unknown<br><br>Why? ………………………………………………...<br>……………………………………………………..
If the driver enters in the storage rooms for the collection of eggs: specific clothes and shoes	□ Always<br>□ Sometimes<br>□ Never<br>□ Other frequency………………………………<br>…………………………………………………….<br>□ Unknown<br>□ Not concerned<br><br>Details: ……………………………………………<br><br>……………………………………………........	□ Too expensive<br>□ Take too much time<br>□ Not enough trained<br>□ Not enough advice<br>□ No knowing advantages<br>□ Not useful<br>□ Not adapted to the farm<br>□ Other reason ………………………………………..<br>……………………………………………………………..<br>□ Unknown<br><br>Details: ………………………………………………	□ Yes<br>□ Moderately<br>□ No<br>□ Other opinion…………………………………<br>……………………………………………………..<br>□ Unknown<br><br>Why? ………………………………………………...<br>……………………………………………………..
Cleaning and disinfection of the storage room after each collection	□ Always<br>□ Sometimes<br>□ Never<br>□ Other frequency………………………………<br>…………………………………………………….<br>□ Unknown<br>□ Not concerned<br><br>Details: ……………………………………………<br><br>……………………………………………........	□ Too expensive<br>□ Take too much time<br>□ Not enough trained<br>□ Not enough advice<br>□ No knowing advantages<br>□ Not useful<br>□ Not adapted to the farm<br>□ Other reason ………………………………………..<br>……………………………………………………………..<br>□ Unknown<br>Details: ………………………………………………	□ Yes<br>□ Moderately<br>□ No<br>□ Other opinion…………………………………<br>……………………………………………………..<br>□ Unknown<br><br>Why? ………………………………………………...<br>……………………………………………………..
Different personal between the poultry room and the eggs sorting and storage room	□ Always<br>□ Sometimes<br>□ Never<br>□ Other frequency………………………………<br>…………………………………………………….<br>□ Unknown<br>□ Not concerned<br><br>Details: ……………………………………………<br><br>……………………………………………........	□ Too expensive<br>□ Take too much time<br>□ Not enough trained<br>□ Not enough advice<br>□ No knowing advantages<br>□ Not useful<br>□ Not adapted to the farm<br>□ Other reason ………………………………………..<br>……………………………………………………………..<br>□ Unknown<br>Details: ………………………………………………	□ Yes<br>□ Moderately<br>□ No<br>□ Other opinion…………………………………<br>……………………………………………………..<br>□ Unknown<br><br>Why? ………………………………………………...<br>……………………………………………………..

Section E. Other Biosecurity measures not always implemented on the farm – All the poultry

**Are there other biosecurity measures that are advice to you but that you not always implement on your farm? ☐ Yes ☐ No**		
	**Could you explain the reasons?**	**Do you think that this measure would be an efficient biosecurity measure for your farm and why?**
**Measure 1** …………………………………………...<br>…………………………………………………………<br>…………………………………………………………	☐ Too expensive<br>☐ Take too much time<br>☐ Not enough trained<br>☐ Not enough advice<br>☐ No knowing advantages<br>☐ Not useful<br>☐ Not adapted to the farm<br>☐ Unknown<br>☐ Other reason ……………………………………………………<br>………………………………………………………………………<br><br>Details : ……………………………………………………….<br>……………………………………………………………………….	☐ Yes<br>☐ Moderately<br>☐ No<br>☐ Unknown<br>☐ Other opinion …………………………………………………<br>……………………………………………………………………….<br><br>Why? ……………………………………………………………..<br>…………………………………………………………………………
**Measure 2** …………………………………………...<br>…………………………………………………………<br>…………………………………………………………	☐ Too expensive<br>☐ Take too much time<br>☐ Not enough trained<br>☐ Not enough advice<br>☐ No knowing advantages<br>☐ Not useful<br>☐ Not adapted to the farm<br>☐ Unknown<br>☐ Other reason ……………………………………………………<br>………………………………………………………………………<br><br>Details : ……………………………………………………….<br>……………………………………………………………………….	☐ Yes<br>☐ Moderately<br>☐ No<br>☐ Unknown<br>☐ Other opinion …………………………………………………<br>……………………………………………………………………….<br><br>Why? ……………………………………………………………..<br>…………………………………………………………………………
**Measure 3** …………………………………………...<br>…………………………………………………………<br>…………………………………………………………	☐ Too expensive<br>☐ Take too much time<br>☐ Not enough trained<br>☐ Not enough advice<br>☐ No knowing advantages<br>☐ Not useful<br>☐ Not adapted to the farm<br>☐ Unknown<br>☐ Other reason ……………………………………………………<br>………………………………………………………………………<br>Details : ……………………………………………………….<br>……………………………………………………………………….	☐ Yes<br>☐ Moderately<br>☐ No<br>☐ Unknown<br>☐ Other opinion …………………………………………………<br>……………………………………………………………………….<br><br>Why? ……………………………………………………………..<br>…………………………………………………………………………

**Measure 4** …………………………………………...<br>…………………………………………………………<br>…………………………………………………………	☐ Too expensive<br>☐ Take too much time<br>☐ Not enough trained<br>☐ Not enough advice<br>☐ No knowing advantages<br>☐ Not useful<br>☐ Not adapted to the farm<br>☐ Unknown<br>☐ Other reason ……………………………………………………<br>………………………………………………………………………<br>Details : ………………………………………………………<br>……………………………………………………………………	☐ Yes<br>☐ Moderately<br>☐ No<br>☐ Unknown<br>☐ Other opinion …………………………………………………<br>…………………………………………………………………………<br><br>Why? ……………………………………………………………...<br>………………………………………………………………………………
**Measure 5** …………………………………………...<br>…………………………………………………………<br>…………………………………………………………	☐ Too expensive<br>☐ Take too much time<br>☐ Not enough trained<br>☐ Not enough advice<br>☐ No knowing advantages<br>☐ Not useful<br>☐ Not adapted to the farm<br>☐ Unknown<br>☐ Other reason ……………………………………………………<br>………………………………………………………………………<br>Details : ………………………………………………………<br>……………………………………………………………………	☐ Yes<br>☐ Moderately<br>☐ No<br>☐ Unknown<br>☐ Other opinion …………………………………………………<br>…………………………………………………………………………<br><br>Why? ……………………………………………………………...<br>………………………………………………………………………………
**Measure 6** …………………………………………...<br>…………………………………………………………<br>…………………………………………………………	☐ Too expensive<br>☐ Take too much time<br>☐ Not enough trained<br>☐ Not enough advice<br>☐ No knowing advantages<br>☐ Not useful<br>☐ Not adapted to the farm<br>☐ Unknown<br>☐ Other reason ……………………………………………………<br>………………………………………………………………………<br>Details : ………………………………………………………<br>……………………………………………………………………	☐ Yes<br>☐ Moderately<br>☐ No<br>☐ Unknown<br>☐ Other opinion …………………………………………………<br>…………………………………………………………………………<br><br>Why? ……………………………………………………………...<br>………………………………………………………………………………

Section F. Successful and Required Supporting measures – All the poultry

**Item S1 - Biosecurity trainings**	**Which successful supporting measures have already helped you to implement biosecurity measures on your farm?**	**Which required supporting measures you would need to improve the implementation of the biosecurity measures on your farm?**	**In your opinion, why do you think that these Supporting measures that you have mentioned here (Successful and required) are effective to improve biosecurity on your farm?**
exposure visit at well-organized farm/field trip	☐<br>Details ……………………………………………<br>………………………………………………………	☐<br>Details ……………………………………………<br>………………………………………………………	………………………………………………<br>………………………………………………
group discussion	☐<br>Details ……………………………………………<br>………………………………………………………	☐<br>Details ……………………………………………<br>………………………………………………………	………………………………………………<br>………………………………………………
live workshops	☐<br>Details ……………………………………………<br>………………………………………………………	☐<br>Details ……………………………………………<br>………………………………………………………	………………………………………………<br>………………………………………………
videos	☐<br>Details ……………………………………………<br>………………………………………………………	☐<br>Details ……………………………………………<br>………………………………………………………	………………………………………………<br>………………………………………………
webinars	☐<br>Details ……………………………………………<br>………………………………………………………	☐<br>Details ……………………………………………<br>………………………………………………………	………………………………………………<br>………………………………………………
educational modules	☐<br>Details ……………………………………………<br>………………………………………………………	☐<br>Details ……………………………………………<br>………………………………………………………	………………………………………………<br>………………………………………………

Other measure 1 in this item<br>…………………………………………	☐<br>Details ……………………………………………<br>…………………………………………………………	☐<br>Details ……………………………………………<br>…………………………………………………………	……………………………………………………<br>……………………………………………………
Other measure 2 in this item<br>…………………………………………	☐<br>Details ……………………………………………<br>…………………………………………………………	☐<br>Details ……………………………………………<br>…………………………………………………………	……………………………………………………<br>……………………………………………………
Other measure 3 in this item<br>…………………………………………	☐<br>Details ……………………………………………<br>…………………………………………………………	☐<br>Details ……………………………………………<br>…………………………………………………………	……………………………………………………<br>……………………………………………………
**Item S2 - Conducting information campaigns promoting biosecurity**	**Which successful supporting measures have already helped you to implement biosecurity measures on your farm?**	**Which required supporting measures you would need to improve the implementation of the biosecurity measures on your farm?**	**In your opinion, why do you think that these Supporting measures that you have mentioned here (Successful and required) are effective to improve biosecurity on your farm?**
conferences/webinars farmer meeting	☐<br>Details ……………………………………………<br>…………………………………………………………	☐<br>Details ……………………………………………<br>…………………………………………………………	……………………………………………………<br>……………………………………………………
Leaflets/banners/posters	☐<br>Details ……………………………………………<br>…………………………………………………………	☐<br>Details ……………………………………………<br>…………………………………………………………	……………………………………………………<br>……………………………………………………
media (TV and web: Youtube etc)	☐<br>Details ……………………………………………<br>…………………………………………………………	☐<br>Details ……………………………………………<br>…………………………………………………………	……………………………………………………<br>……………………………………………………
social media groups (Facebook, LinkedIn, etc)	☐<br>Details ……………………………………………<br>…………………………………………………………	☐<br>Details ……………………………………………<br>…………………………………………………………	……………………………………………………<br>……………………………………………………

Gadgets: (lines, pencils, key rings, cups, bags etc.)	☐<br>Details ……………………………………………<br>………………………………………………………	☐<br>Details ……………………………………………<br>………………………………………………………	………………………………………………………<br>………………………………………………………
Other measure 1 in this item<br>………………………………………….	☐<br>Details ……………………………………………<br>………………………………………………………	☐<br>Details ……………………………………………<br>………………………………………………………	………………………………………………………<br>………………………………………………………
Other measure 2 in this item<br>………………………………………….	☐<br>Details ……………………………………………<br>………………………………………………………	☐<br>Details ……………………………………………<br>………………………………………………………	………………………………………………………<br>………………………………………………………
Other measure 3 in this item<br>………………………………………….	☐<br>Details ……………………………………………<br>………………………………………………………	☐<br>Details ……………………………………………<br>………………………………………………………	………………………………………………………<br>………………………………………………………
**Item S3 - Educational material**	**Which successful supporting measures have already helped you to implement biosecurity measures on your farm?**	**Which required supporting measures you would need to improve the implementation of the biosecurity measures on your farm?**	**In your opinion, why do you think that these Supporting measures that you have mentioned here (Successful and required) are effective to improve biosecurity on your farm?**
Books/guides/manuals/ research papers/journals/ farming press	☐<br>Details ……………………………………………<br>………………………………………………………	☐<br>Details ……………………………………………<br>………………………………………………………	………………………………………………………<br>………………………………………………………
Posters/banners/newsletters /leaflets	☐<br>Details ……………………………………………<br>………………………………………………………	☐<br>Details ……………………………………………<br>………………………………………………………	………………………………………………………<br>………………………………………………………
Media: TV and web (YouTube etc.)	☐<br>Details ……………………………………………<br>………………………………………………………	☐<br>Details ……………………………………………<br>………………………………………………………	………………………………………………………<br>………………………………………………………

social media groups (Facebook, LinkedIn, etc)	☐<br>Details ……………………………………………<br>……………………………………………………….	☐<br>Details ……………………………………………<br>……………………………………………………….	………………………………………………….<br>………………………………………………….
Other measure 1 in this item<br>………………………………………….	☐<br>Details ……………………………………………<br>……………………………………………………….	☐<br>Details ……………………………………………<br>……………………………………………………….	………………………………………………….<br>………………………………………………….
Other measure 2 in this item<br>………………………………………….	☐<br>Details ……………………………………………<br>……………………………………………………….	☐<br>Details ……………………………………………<br>……………………………………………………….	………………………………………………….<br>………………………………………………….
Other measure 3 in this item<br>………………………………………….	☐<br>Details ……………………………………………<br>……………………………………………………….	☐<br>Details ……………………………………………<br>……………………………………………………….	………………………………………………….<br>………………………………………………….
**Item S4 - Biosecurity checks (audits)**	**Which successful supporting measures have already helped you to implement biosecurity measures on your farm?**	**Which required supporting measures you would need to improve the implementation of the biosecurity measures on your farm?**	**In your opinion, why do you think that these Supporting measures that you have mentioned here (Successful and required) are effective to improve biosecurity on your farm?**
by government	☐<br>Details ……………………………………………<br>……………………………………………………….	☐<br>Details ……………………………………………<br>……………………………………………………….	………………………………………………….<br>………………………………………………….
by stakeholders (local integration companies etc.)	☐<br>Details ……………………………………………<br>……………………………………………………….	☐<br>Details ……………………………………………<br>……………………………………………………….	………………………………………………….<br>………………………………………………….

Other measure 1 in this item<br>…………………………………………	☐<br>Details ……………………………………………<br>………………………………………………………	☐<br>Details ……………………………………………<br>………………………………………………………	………………………………………………<br>………………………………………………
Other measure 2 in this item<br>…………………………………………	☐<br>Details ……………………………………………<br>………………………………………………………	☐<br>Details ……………………………………………<br>………………………………………………………	………………………………………………<br>………………………………………………
Other measure 3 in this item<br>…………………………………………	☐<br>Details ……………………………………………<br>………………………………………………………	☐<br>Details ……………………………………………<br>………………………………………………………	………………………………………………<br>………………………………………………
**Item S5- Regulations set up supporting biosecurity implementation**	**Which successful supporting measures have already helped you to implement biosecurity measures on your farm?**	**Which required supporting measures you would need to improve the implementation of the biosecurity measures on your farm?**	**In your opinion, why do you think that these Supporting measures that you have mentioned here (Successful and required) are effective to improve biosecurity on your farm?**
Regulations set up supporting biosecurity implementation	☐<br>Details ……………………………………………<br>………………………………………………………	☐<br>Details ……………………………………………<br>………………………………………………………	………………………………………………<br>………………………………………………
Other measure 1 in this item<br>…………………………………………	☐<br>Details ……………………………………………<br>………………………………………………………	☐<br>Details ……………………………………………<br>………………………………………………………	………………………………………………<br>………………………………………………
Other measure 2 in this item<br>…………………………………………	☐<br>Details ……………………………………………<br>………………………………………………………	☐<br>Details ……………………………………………<br>………………………………………………………	………………………………………………<br>………………………………………………
Other measure 3 in this item<br>…………………………………………	☐<br>Details ……………………………………………<br>………………………………………………………	☐<br>Details ……………………………………………<br>………………………………………………………	………………………………………………<br>………………………………………………

Item S6- Support by a biosecurity advisor (coach /vets)	Which successful supporting measures have already helped you to implement biosecurity measures on your farm?	Which required supporting measures you would need to improve the implementation of the biosecurity measures on your farm?	In your opinion, why do you think that these Supporting measures that you have mentioned here (Successful and required) are effective to improve biosecurity on your farm?
Contact support (farm visiting)	☐<br>Details ………………………………………………<br>…………………………………………………………	☐<br>Details ………………………………………………<br>…………………………………………………………	……………………………………………………<br>……………………………………………………
distance support (by phone, email, Facebook etc.)	☐<br>Details ………………………………………………<br>…………………………………………………………	☐<br>Details ………………………………………………<br>…………………………………………………………	……………………………………………………<br>……………………………………………………
Farmer coaching methods	☐<br>Details ………………………………………………<br>…………………………………………………………	☐<br>Details ………………………………………………<br>…………………………………………………………	……………………………………………………<br>……………………………………………………
Other measure 1 in this item<br>……………………………………………	☐<br>Details ………………………………………………<br>…………………………………………………………	☐<br>Details ………………………………………………<br>…………………………………………………………	……………………………………………………<br>……………………………………………………
Other measure 2 in this item<br>……………………………………………	☐<br>Details ………………………………………………<br>…………………………………………………………	☐<br>Details ………………………………………………<br>…………………………………………………………	……………………………………………………<br>……………………………………………………
Other measure 3 in this item<br>……………………………………………	☐<br>Details ………………………………………………<br>…………………………………………………………	☐<br>Details ………………………………………………<br>…………………………………………………………	……………………………………………………<br>……………………………………………………

**Item S7 - Organisation of competition for best biosecurity (eg “biosecurity award”)**	**Which successful supporting measures have already helped you to implement biosecurity measures on your farm?**	**Which required supporting measures you would need to improve the implementation of the biosecurity measures on your farm?**	**In your opinion, why do you think that these Supporting measures that you have mentioned here (Successful and required) are effective to improve biosecurity on your farm?**
Organisation of competition for best biosecurity (eg “biosecurity award”)	☐<br>Details ……………………………………………<br>………………………………………………………	☐<br>Details ……………………………………………<br>………………………………………………………	………………………………………………<br>………………………………………………
Other measure 1 in this item<br>…………………………………………	☐<br>Details ……………………………………………<br>………………………………………………………	☐<br>Details ……………………………………………<br>………………………………………………………	………………………………………………<br>………………………………………………
Other measure 2 in this item<br>…………………………………………	☐<br>Details ……………………………………………<br>………………………………………………………<br>.	☐<br>Details ……………………………………………<br>………………………………………………………	………………………………………………<br>………………………………………………
Other measure 3 in this item<br>…………………………………………	☐<br>Details ……………………………………………<br>………………………………………………………	☐<br>Details ……………………………………………<br>………………………………………………………	………………………………………………<br>………………………………………………
**Item S8 - Financial support for biosecurity implementation**	**Which successful supporting measures have already helped you to implement biosecurity measures on your farm?**	**Which required supporting measures you would need to improve the implementation of the biosecurity measures on your farm?**	**In your opinion, why do you think that these Supporting measures that you have mentioned here (Successful and required) are effective to improve biosecurity on your farm?**
Financial support for biosecurity implementation	☐<br>Details ……………………………………………<br>………………………………………………………	☐<br>Details ……………………………………………<br>………………………………………………………	………………………………………………<br>………………………………………………

Other measure 1 in this item<br>…………………………………………	☐<br>Details ……………………………………………<br>……………………………………………………………	☐<br>Details ……………………………………………<br>……………………………………………………………	……………………………………………………<br>……………………………………………………
Other measure 2 in this item<br>…………………………………………	☐<br>Details ……………………………………………<br>……………………………………………………………	☐<br>Details ……………………………………………<br>……………………………………………………………	……………………………………………………<br>……………………………………………………
Other measure 3 in this item<br>…………………………………………	☐<br>Details ……………………………………………<br>……………………………………………………………	☐<br>Details ……………………………………………<br>……………………………………………………………	……………………………………………………<br>……………………………………………………
**Item S9 - Other supporting measures (not described above)**	**Which successful supporting measures have already helped you to implement biosecurity measures on your farm?**	**Which required supporting measures you would need to improve the implementation of the biosecurity measures on your farm?**	**In your opinion, why do you think that these Supporting measures that you have mentioned here (Successful and required) are effective to improve biosecurity on your farm?**
Other measure 1<br>…………………………………………	☐<br>Details ……………………………………………<br>……………………………………………………………	☐<br>Details ……………………………………………<br>……………………………………………………………	……………………………………………………<br>……………………………………………………
Other measure 2<br>…………………………………………	☐<br>Details ……………………………………………<br>……………………………………………………………	☐<br>Details ……………………………………………<br>……………………………………………………………	……………………………………………………<br>……………………………………………………
Other measure 3<br>…………………………………………	☐<br>Details ……………………………………………<br>……………………………………………………………	☐<br>Details ……………………………………………<br>……………………………………………………………	……………………………………………………<br>……………………………………………………
Other measure 4<br>…………………………………………	☐<br>Details ……………………………………………<br>……………………………………………………………	☐<br>Details ……………………………………………<br>……………………………………………………………	……………………………………………………<br>……………………………………………………

Other measure 5<br>…………………………………………	☐<br>Details ……………………………………………<br>………………………………………………………	☐<br>Details ……………………………………………<br>………………………………………………………	…………………………………………………<br>…………………………………………………
Other measure 6<br>…………………………………………	☐<br>Details ……………………………………………<br>………………………………………………………	☐<br>Details ……………………………………………<br>………………………………………………………	…………………………………………………<br>…………………………………………………
