# Supplementary material for: Stakeholders’ Perceptions of Biosecurity Implementation in Italian Poultry Farms
Source: Animals (Basel). 2023 Oct 18;13(20):3246. doi: 10.3390/ani13203246 (PMC10603624; doi:10.3390/ani13203246)
Supplement: Supplementary file 1 [file animals-13-03246-s001.zip › Table S2_Advisor questionnaire.pdf]

Questionnaire ADVISER – NETPOULSAFE (Final – 05/05/2021)

Objectives and structure of the questionnaire

Objectives of the interview:
- to collect the opinion of the advisers on the implementation of the **biosecurity measures** on the farms, the reasons explaining the difficulties to implement them and their opinion on the efficiency.
- to collect the opinion of the advisers on the **supporting measures** that have already helped the farmers to improve the biosecurity on their farm (successful measures) or that the farmers would need (required measures), with the reasons for the measures not implemented and the solutions.

Structure of the questionnaire:

Part 1: Implementation of the Biosecurity Measures on the farms and the reasons of difficulties

Section A: Similar Biosecurity items - **All the poultry (12 items)**
Section B: Supplementary Biosecurity item - **Free range (1 item)**
Section C: Supplementary Biosecurity item - **Breeder (1 item)**
Section D: Supplementary Biosecurity item - **Layer (1 item)**
Section E: Other Biosecurity measures not always implemented on the farms - **All the poultry**

Part 2: Use of the Supporting Measures by the farmers to improve biosecurity on their farm

Section F: Successful and Required Supporting measures - **All the poultry (9 items)**

***(Item E:** External biosecurity measures, **Item I:** Internal biosecurity measures**, Item S:** Supporting biosecurity measures)*

Interviewer : Network Facilitator

**Country:** ☐ **BE Belgium** ☐ **ES Spain** ☐ **FR France** ☐ **HU Hungary** ☐ **IT Italy** ☐ **NL Netherlands** ☐ **PL Poland**	Date of the interview: ………………………………..

Adviser questioned

N° Adviser (1 to X): …………

Gender ☐ man ☐ woman Age category ☐ <35 ☐ 35-55 ☐ >55

☐ Producers organization (interprofession/quality label/other organization)
☐ Companies (production)
☐ Vets (organization or practician)
☐ Local public institutions
☐ Other ………
Details: ………

The Poultry production questioned (1 poultry production)

☐ **Enclosed Broilers** ☐ **Enclosed Turkeys** ☐ **Enclosed ducks** ☐ **Enclosed layers**
☐ **Free range Broilers** ☐ **Free range Ducks** ☐ **Free range layers** ☐ **Breeders**
☐ **Other …………………………………………**
Details: ………

Farming system	☐ Independent for direct sales<br>☐ Under contract with a company for product sales<br>☐ Other ………………………………………….
Type of production	☐ Standard<br>☐ Organic<br>☐ Label<br>☐ Other ……………………………………………

Section A: Similar Biosecurity items - All the poultry

**Item E1 - Animal production on the site**	**According to you, does this measure implemented on the farms?**	**For a measure not always implemented on the farms**	
		**Could you explain the reasons?**	**Do you think that this measure would be an efficient biosecurity measure for the farms and why?**
"all-in/all-out" poultry production on the site	☐ Always<br>☐ Sometimes<br>☐ Never<br>☐ Other frequency………………………………….<br>…………………………………………………………..<br>☐ Unknown<br>☐ Not concerned<br><br>Details: ……………………………………………<br>…………………………………………………….........	☐ Too expensive<br>☐ Take too much time<br>☐ Not enough trained<br>☐ Not enough advice<br>☐ No knowing advantages<br>☐ Not useful<br>☐ Not adapted to the farm<br>☐ Other reason ……………………………………….<br>……………………………………………………………..<br>☐ Unknown<br><br>Details: ……………………………………………….<br>…………………………………………………………..	☐ Yes<br>☐ Moderately<br>☐ No<br>☐ Other opinion…………………………………….<br>…………………………………………………………..<br>☐ Unknown<br><br>Why? ………………………………………………….<br>……………………………………………………………..
No backyard on the site	☐ Always<br>☐ Sometimes<br>☐ Never<br>☐ Other frequency………………………………….<br>…………………………………………………………..<br>☐ Unknown<br>☐ Not concerned<br><br>Details: ……………………………………………<br><br>…………………………………………………….........	☐ Too expensive<br>☐ Take too much time<br>☐ Not enough trained<br>☐ Not enough advice<br>☐ No knowing advantages<br>☐ Not useful<br>☐ Not adapted to the farm<br>☐ Other reason ……………………………………….<br>……………………………………………………………..<br>☐ Unknown<br><br>Details: ……………………………………………….<br>………………………………………………………….	☐ Yes<br>☐ Moderately<br>☐ No<br>☐ Other opinion…………………………………….<br>…………………………………………………………..<br>☐ Unknown<br><br>Why? ………………………………………………...<br>…………………………………………………………..
If other animal productions on the site (cattle, pigs) sanitary barriers with poultry (personal, material …)	☐ Always<br>☐ Sometimes<br>☐ Never<br>☐ Other frequency………………………………….<br>…………………………………………………………..<br>☐ Unknown<br>☐ Not concerned<br><br>Details: ……………………………………………<br>…………………………………………………….........	☐ Too expensive<br>☐ Take too much time<br>☐ Not enough trained<br>☐ Not enough advice<br>☐ No knowing advantages<br>☐ Not useful<br>☐ Not adapted to the farm<br>☐ Other reason ……………………………………….<br>……………………………………………………………..<br>☐ Unknown<br><br>Details: ……………………………………………….<br>…………………………………………………………..	☐ Yes<br>☐ Moderately<br>☐ No<br>☐ Other opinion…………………………………….<br>…………………………………………………………..<br>☐ Unknown<br><br>Why? ………………………………………………….<br>…………………………………………………………..

Item E2 - Structure and circulation on the site	According to you, does this measure implemented on the farms?	For a measure not always implemented on the farms	
		Could you explain the reasons?	Do you think that this measure would be an efficient biosecurity measure for the farms and why?
Delimitation with a barrier or closure of a professional secured area with only necessary vehicles to the poultry house (feed, chicks, poultry or eggs transport vehicles)	☐ Always<br>☐ Sometimes<br>☐ Never<br>☐ Other frequency…………………………………<br>……………………………………………………………<br>☐ Unknown<br>☐ Not concerned<br><br>Details: ……………………………………………<br><br>…………………………………………………….......	☐ Too expensive<br>☐ Take too much time<br>☐ Not enough trained<br>☐ Not enough advice<br>☐ No knowing advantages<br>☐ Not useful<br>☐ Not adapted to the farm<br>☐ Other reason ……………………………………….<br>…………………………………………………………..<br>☐ Unknown<br><br>Details: ……………………………………………….<br><br>………………………………………………………….	☐ Yes<br>☐ Moderately<br>☐ No<br>☐ Other opinion……………………………………<br>……………………………………………………………<br>☐ Unknown<br><br>Why? ………………………………………………<br>………………………………………………………….
Wheel dips for disinfection of the vehicles or pulverization before entering on the site	☐ Always<br>☐ Sometimes<br>☐ Never<br>☐ Other frequency…………………………………<br>……………………………………………………………<br>☐ Unknown<br>☐ Not concerned<br><br>Details: ……………………………………………<br><br>…………………………………………………….......	☐ Too expensive<br>☐ Take too much time<br>☐ Not enough trained<br>☐ Not enough advice<br>☐ No knowing advantages<br>☐ Not useful<br>☐ Not adapted to the farm<br>☐ Other reason ……………………………………….<br>…………………………………………………………..<br>☐ Unknown<br><br>Details: ………………………………………………<br><br>…………………………………………………………	☐ Yes<br>☐ Moderately<br>☐ No<br>☐ Other opinion………………………………….<br>………………………………………………………….<br>☐ Unknown<br><br>Why? ……………………………………………...<br>……………………………………………………….
**Item E3 - Personnel, visitors or teams**	**According to you, does this measure implemented on the farms?**	**For a measure not always implemented on the farms**	
		**Could you explain the reasons?**	**Do you think that this measure would be an efficient biosecurity measure for the farms and why?**
**Personnel**			
Specific clothes before entering in the house	☐ Always<br>☐ Sometimes<br>☐ Never<br>☐ Other frequency…………………………………<br>……………………………………………………………<br>☐ Unknown<br>☐ Not concerned<br><br>Details: ……………………………………………<br><br>…………………………………………………….......	☐ Too expensive<br>☐ Take too much time<br>☐ Not enough trained<br>☐ Not enough advice<br>☐ No knowing advantages<br>☐ Not useful<br>☐ Not adapted to the farm<br>☐ Other reason ……………………………………….<br>…………………………………………………………..<br>☐ Unknown<br>Details: ………………………………………………	☐ Yes<br>☐ Moderately<br>☐ No<br>☐ Other opinion……………………………………<br>……………………………………………………………<br>☐ Unknown<br><br>Why? ………………………………………………<br>………………………………………………………….

Specific shoes before entering in the house	☐ Always<br>☐ Sometimes<br>☐ Never<br>☐ Other frequency…………………………………<br>……………………………………………………….<br>☐ Unknown<br>☐ Not concerned<br><br>Details: ……………………………………………<br><br>………………………………………………….......	☐ Too expensive<br>☐ Take too much time<br>☐ Not enough trained<br>☐ Not enough advice<br>☐ No knowing advantages<br>☐ Not useful<br>☐ Not adapted to the farm<br>☐ Other reason ……………………………………….<br>………………………………………………………..<br>☐ Unknown<br>Details: ………………………………………………	☐ Yes<br>☐ Moderately<br>☐ No<br>☐ Other opinion………………………………….<br>……………………………………………………….<br>☐ Unknown<br><br>Why? ………………………………………………...<br>………………………………………………………..
Washing of the hands before entering in the house	☐ Always<br>☐ Sometimes<br>☐ Never<br>☐ Other frequency…………………………………<br>……………………………………………………….<br>☐ Unknown<br>☐ Not concerned<br><br>Details: ……………………………………………<br><br>………………………………………………….......	☐ Too expensive<br>☐ Take too much time<br>☐ Not enough trained<br>☐ Not enough advice<br>☐ No knowing advantages<br>☐ Not useful<br>☐ Not adapted to the farm<br>☐ Other reason ……………………………………….<br>………………………………………………………..<br>☐ Unknown<br><br>Details: ………………………………………………	☐ Yes<br>☐ Moderately<br>☐ No<br>☐ Other opinion……………………………………<br>…………………………………………………………<br>☐ Unknown<br><br>Why? ………………………………………………<br>………………………………………………………….
Showering before entering in the house	☐ Always<br>☐ Sometimes<br>☐ Never<br>☐ Other frequency…………………………………<br>……………………………………………………….<br>☐ Unknown<br>☐ Not concerned<br><br>Details: ……………………………………………<br><br>………………………………………………….......	☐ Too expensive<br>☐ Take too much time<br>☐ Not enough trained<br>☐ Not enough advice<br>☐ No knowing advantages<br>☐ Not useful<br>☐ Not adapted to the farm<br>☐ Other reason ……………………………………….<br>………………………………………………………..<br>☐ Unknown<br>Details: ………………………………………………	☐ Yes<br>☐ Moderately<br>☐ No<br>☐ Other opinion……………………………………<br>…………………………………………………………<br>☐ Unknown<br><br>Why? ………………………………………………<br>………………………………………………………….
**Visitors or teams**			
Register for visitors and teams	☐ Always<br>☐ Sometimes<br>☐ Never<br>☐ Other frequency…………………………………<br>……………………………………………………….<br>☐ Unknown<br>☐ Not concerned<br><br>Details: ……………………………………………<br><br>………………………………………………….......	☐ Too expensive<br>☐ Take too much time<br>☐ Not enough trained<br>☐ Not enough advice<br>☐ No knowing advantages<br>☐ Not useful<br>☐ Not adapted to the farm<br>☐ Other reason ……………………………………….<br>………………………………………………………..<br>☐ Unknown<br>Details: ………………………………………………	☐ Yes<br>☐ Moderately<br>☐ No<br>☐ Other opinion……………………………………<br>…………………………………………………………<br>☐ Unknown<br><br>Why? ………………………………………………<br>………………………………………………………….

Specific clothes before entering in the house	☐ Always<br>☐ Sometimes<br>☐ Never<br>☐ Other frequency…………………………………<br>……………………………………………………….<br>☐ Unknown<br>☐ Not concerned<br><br>Details: ……………………………………………<br><br>………………………………………………….…...	☐ Too expensive<br>☐ Take too much time<br>☐ Not enough trained<br>☐ Not enough advice<br>☐ No knowing advantages<br>☐ Not useful<br>☐ Not adapted to the farm<br>☐ Other reason ……………………………………..<br>…………………………………………………………..<br>☐ Unknown<br>Details: ……………………………………………….	☐ Yes<br>☐ Moderately<br>☐ No<br>☐ Other opinion…………………………………….<br>………………………………………………………..<br>☐ Unknown<br><br>Why? ……………………………………………...<br>………………………………………………………..
Specific shoes before entering in the house	☐ Always<br>☐ Sometimes<br>☐ Never<br>☐ Other frequency…………………………………<br>……………………………………………………….<br>☐ Unknown<br>☐ Not concerned<br><br>Details: ……………………………………………<br><br>………………………………………………….…...	☐ Too expensive<br>☐ Take too much time<br>☐ Not enough trained<br>☐ Not enough advice<br>☐ No knowing advantages<br>☐ Not useful<br>☐ Not adapted to the farm<br>☐ Other reason ……………………………………..<br>…………………………………………………………..<br>☐ Unknown<br>Details: ……………………………………………….	☐ Yes<br>☐ Moderately<br>☐ No<br>☐ Other opinion……………………………………..<br>………………………………………………………….<br>☐ Unknown<br><br>Why? ………………………………………………<br>………………………………………………………….
Washing of the hands before entering in the house	☐ Always<br>☐ Sometimes<br>☐ Never<br>☐ Other frequency…………………………………<br>……………………………………………………….<br>☐ Unknown<br>☐ Not concerned<br><br>Details: ……………………………………………<br><br>………………………………………………….…...	☐ Too expensive<br>☐ Take too much time<br>☐ Not enough trained<br>☐ Not enough advice<br>☐ No knowing advantages<br>☐ Not useful<br>☐ Not adapted to the farm<br>☐ Other reason ……………………………………..<br>…………………………………………………………..<br>☐ Unknown<br>Details: ……………………………………………….	☐ Yes<br>☐ Moderately<br>☐ No<br>☐ Other opinion……………………………………..<br>………………………………………………………….<br>☐ Unknown<br><br>Why? ………………………………………………<br>………………………………………………………….
Showering before entering in the house	☐ Always<br>☐ Sometimes<br>☐ Never<br>☐ Other frequency…………………………………<br>……………………………………………………….<br>☐ Unknown<br>☐ Not concerned<br><br>Details: ……………………………………………<br><br>………………………………………………….…...	☐ Too expensive<br>☐ Take too much time<br>☐ Not enough trained<br>☐ Not enough advice<br>☐ No knowing advantages<br>☐ Not useful<br>☐ Not adapted to the farm<br>☐ Other reason ……………………………………..<br>…………………………………………………………..<br>☐ Unknown<br>Details: ……………………………………………….<br><br>………………………………………………………….	☐ Yes<br>☐ Moderately<br>☐ No<br>☐ Other opinion……………………………………..<br>………………………………………………………….<br>☐ Unknown<br><br>Why? ………………………………………………<br>………………………………………………………….

**Item E4 - The poultry at the arrival**	**According to you, does this measure implemented on the farms?**	**For a measure not always implemented on the farms**	
		**Could you explain the reasons?**	**Do you think that this measure would be an efficient biosecurity measure for the farms and why?**
Register for the flock (origin, number of poultry, …)	□ Always<br>□ Sometimes<br>□ Never<br>□ Other frequency…………………………………<br>…………………………………………………………..<br>□ Unknown<br>□ Not concerned<br><br>Details: ……………………………………………<br><br>……………………………………………………........	□ Too expensive<br>□ Take too much time<br>□ Not enough trained<br>□ Not enough advice<br>□ No knowing advantages<br>□ Not useful<br>□ Not adapted to the farm<br>□ Other reason ………………………………………..<br>…………………………………………………………..<br>□ Unknown<br>Details: ……………………………………………….<br><br>………………………………………………………….	□ Yes<br>□ Moderately<br>□ No<br>□ Other opinion…………………………………….<br>…………………………………………………………<br>□ Unknown<br><br>Why? ………………………………………………<br>………………………………………………………….
If the chicks deliverer enters in the house: specific clothes and shoes	□ Always<br>□ Sometimes<br>□ Never<br>□ Other frequency…………………………………<br>…………………………………………………………..<br>□ Unknown<br>□ Not concerned<br><br>Details: ……………………………………………<br><br>……………………………………………………........	□ Too expensive<br>□ Take too much time<br>□ Not enough trained<br>□ Not enough advice<br>□ No knowing advantages<br>□ Not useful<br>□ Not adapted to the farm<br>□ Other reason ………………………………………..<br>…………………………………………………………..<br>□ Unknown<br>Details: ……………………………………………….<br><br>………………………………………………………….	□ Yes<br>□ Moderately<br>□ No<br>□ Other opinion…………………………………….<br>…………………………………………………………..<br>□ Unknown<br><br>Why? ………………………………………………...<br>…………………………………………………………..
**Item E5 - Feed and drinking water of the poultry**	**According to you, does this measure implemented on the farms?**	**For a measure not always implemented on the farms**	
		**Could you explain the reasons?**	**Do you think that this measure would be an efficient biosecurity measure for the farms and why?**
Feed storage protection	□ Always<br>□ Sometimes<br>□ Never<br>□ Other frequency…………………………………<br>…………………………………………………………..<br>□ Unknown<br>□ Not concerned<br><br>Details: ……………………………………………<br><br>……………………………………………………........	□ Too expensive<br>□ Take too much time<br>□ Not enough trained<br>□ Not enough advice<br>□ No knowing advantages<br>□ Not useful<br>□ Not adapted to the farm<br>□ Other reason ………………………………………..<br>…………………………………………………………..<br>□ Unknown<br>Details: ……………………………………………….<br><br>………………………………………………………….	□ Yes<br>□ Moderately<br>□ No<br>□ Other opinion…………………………………….<br>…………………………………………………………<br>□ Unknown<br><br>Why? ………………………………………………<br>………………………………………………………….

Drinking water analysis end line each year	☐ Always<br>☐ Sometimes<br>☐ Never<br>☐ Other frequency…………………………………<br>………………………………………………………..<br>☐ Unknown<br>☐ Not concerned<br><br>Details: ……………………………………………<br><br>…………………………………………………........	☐ Too expensive<br>☐ Take too much time<br>☐ Not enough trained<br>☐ Not enough advice<br>☐ No knowing advantages<br>☐ Not useful<br>☐ Not adapted to the farm<br>☐ Other reason ……………………………………….<br>…………………………………………………………..<br>☐ Unknown<br><br>Details: ……………………………………………….<br><br>…………………………………………………………	☐ Yes<br>☐ Moderately<br>☐ No<br>☐ Other opinion………………………………….<br>………………………………………………………..<br>☐ Unknown<br><br>Why? ………………………………………………...<br>………………………………………………………..
**Item E6 - Biological vectors control**	**According to you, does this measure implemented on the farms?**	**For a measure not always implemented on the farms**<br>**Could you explain the reasons?**	**Do you think that this measure would be an efficient biosecurity measure for the farms and why?**
Rodents control (deratting or other measures)	☐ Always<br>☐ Sometimes<br>☐ Never<br>☐ Other frequency…………………………………<br>………………………………………………………..<br>☐ Unknown<br>☐ Not concerned<br><br>Details: ……………………………………………<br><br>…………………………………………………........	☐ Too expensive<br>☐ Take too much time<br>☐ Not enough trained<br>☐ Not enough advice<br>☐ No knowing advantages<br>☐ Not useful<br>☐ Not adapted to the farm<br>☐ Other reason ……………………………………….<br>…………………………………………………………..<br>☐ Unknown<br><br>Details: ……………………………………………….<br><br>………………………………………………………….	☐ Yes<br>☐ Moderately<br>☐ No<br>☐ Other opinion…………………………………….<br>…………………………………………………………<br>☐ Unknown<br><br>Why? …………………………………………………<br>………………………………………………………….
Wild birds control (protection of the ventilation circuit or other measures)	☐ Always<br>☐ Sometimes<br>☐ Never<br>☐ Other frequency…………………………………<br>………………………………………………………..<br>☐ Unknown<br>☐ Not concerned<br><br>Details: ……………………………………………<br><br>…………………………………………………........	☐ Too expensive<br>☐ Take too much time<br>☐ Not enough trained<br>☐ Not enough advice<br>☐ No knowing advantages<br>☐ Not useful<br>☐ Not adapted to the farm<br>☐ Other reason ……………………………………….<br>…………………………………………………………..<br>☐ Unknown<br><br>Details: ……………………………………………….<br><br>…………………………………………………………	☐ Yes<br>☐ Moderately<br>☐ No<br>☐ Other opinion………………………………….<br>………………………………………………………..<br>☐ Unknown<br><br>Why? ………………………………………………...<br>………………………………………………………..

No domestic animals on the site (pets, dogs or cats)	☐ Always<br>☐ Sometimes<br>☐ Never<br>☐ Other frequency……………………………………<br>……………………………………………………………..<br>☐ Unknown<br>☐ Not concerned<br><br>Details: ……………………………………………<br><br>………………………………………………..........	☐ Too expensive<br>☐ Take too much time<br>☐ Not enough trained<br>☐ Not enough advice<br>☐ No knowing advantages<br>☐ Not useful<br>☐ Not adapted to the farm<br>☐ Other reason ………………………………………..<br>…………………………………………………………..<br>☐ Unknown<br>Details: ………………………………………………<br><br>…………………………………………………………	☐ Yes<br>☐ Moderately<br>☐ No<br>☐ Other opinion……………………………………<br>…………………………………………………………<br>☐ Unknown<br><br>Why? ………………………………………………<br>……………………………………………………….
**Item E7 - Management of the poultry manure**	**According to you, does this measure implemented on the farms?**	**For a measure not always implemented on the farms**	
		**Could you explain the reasons?**	**Do you think that this measure would be an efficient biosecurity measure for the farms and why?**
Manure stored in a specific isolated area outside of the secured professional area (or if no secured area : away from the house)	☐ Always<br>☐ Sometimes<br>☐ Never<br>☐ Other frequency……………………………………<br>……………………………………………………………..<br>☐ Unknown<br>☐ Not concerned<br><br>Details: ……………………………………………<br><br>………………………………………………..........	☐ Too expensive<br>☐ Take too much time<br>☐ Not enough trained<br>☐ Not enough advice<br>☐ No knowing advantages<br>☐ Not useful<br>☐ Not adapted to the farm<br>☐ Other reason ………………………………………..<br>…………………………………………………………..<br>☐ Unknown<br>Details: ………………………………………………<br><br>…………………………………………………………	☐ Yes<br>☐ Moderately<br>☐ No<br>☐ Other opinion……………………………………<br>…………………………………………………………<br>☐ Unknown<br><br>Why? ………………………………………………<br>……………………………………………………….
**Item E8 - Management of dead animals**	**According to you, does this measure implemented on the farms?**	**For a measure not always implemented on the farms**	
		**Could you explain the reasons?**	**Do you think that this measure would be an efficient biosecurity measure for the farms and why?**
Removal of the carcasses at least twice a day	☐ Always<br>☐ Sometimes<br>☐ Never<br>☐ Other frequency……………………………………<br>……………………………………………………………..<br>☐ Unknown<br>☐ Not concerned<br><br>Details: ……………………………………………<br><br>………………………………………………..........	☐ Too expensive<br>☐ Take too much time<br>☐ Not enough trained<br>☐ Not enough advice<br>☐ No knowing advantages<br>☐ Not useful<br>☐ Not adapted to the farm<br>☐ Other reason ………………………………………..<br>…………………………………………………………..<br>☐ Unknown<br><br>Details: ………………………………………………<br><br>…………………………………………………………	☐ Yes<br>☐ Moderately<br>☐ No<br>☐ Other opinion……………………………………<br>…………………………………………………………<br>☐ Unknown<br><br>Why? ………………………………………………<br>……………………………………………………….

Presence of a closed and protected rendering tank	☐ Always<br>☐ Sometimes<br>☐ Never<br>☐ Other frequency…………………………………<br>………………………………………………………..<br>☐ Unknown<br>☐ Not concerned<br><br>Details: ……………………………………………<br><br>………………………………………………..........	☐ Too expensive<br>☐ Take too much time<br>☐ Not enough trained<br>☐ Not enough advice<br>☐ No knowing advantages<br>☐ Not useful<br>☐ Not adapted to the farm<br>☐ Other reason ………………………………………..<br>……………………………………………………………..<br>☐ Unknown<br><br>Details: ………………………………………………<br><br>…………………………………………………………	☐ Yes<br>☐ Moderately<br>☐ No<br>☐ Other opinion…………………………………….<br>………………………………………………………..<br>☐ Unknown<br><br>Why? ………………………………………………...<br>………………………………………………………..
Rendering tank located outside of the secured area (or if no secured area : away from the house) allowing the passage of the truck away from the house	☐ Always<br>☐ Sometimes<br>☐ Never<br>☐ Other frequency…………………………………<br>………………………………………………………..<br>☐ Unknown<br>☐ Not concerned<br><br>Details: ……………………………………………<br><br>………………………………………………..........	☐ Too expensive<br>☐ Take too much time<br>☐ Not enough trained<br>☐ Not enough advice<br>☐ No knowing advantages<br>☐ Not useful<br>☐ Not adapted to the farm<br>☐ Other reason ………………………………………..<br>……………………………………………………………..<br>☐ Unknown<br><br>Details: ………………………………………………<br><br>…………………………………………………………	☐ Yes<br>☐ Moderately<br>☐ No<br>☐ Other opinion……………………………………<br>…………………………………………………………<br>☐ Unknown<br><br>Why? ………………………………………………<br>…………………………………………………………
Cleaning and disinfection of the rendering tank after each collection	☐ Always<br>☐ Sometimes<br>☐ Never<br>☐ Other frequency…………………………………<br>………………………………………………………..<br>☐ Unknown<br>☐ Not concerned<br><br>Details: ……………………………………………<br><br>………………………………………………..........	☐ Too expensive<br>☐ Take too much time<br>☐ Not enough trained<br>☐ Not enough advice<br>☐ No knowing advantages<br>☐ Not useful<br>☐ Not adapted to the farm<br>☐ Other reason ………………………………………..<br>……………………………………………………………..<br>☐ Unknown<br><br>Details: ………………………………………………<br><br>…………………………………………………………	☐ Yes<br>☐ Moderately<br>☐ No<br>☐ Other opinion……………………………………<br>…………………………………………………………<br>☐ Unknown<br><br>Why? ………………………………………………<br>…………………………………………………………

**Item I1 - Structure and circulation in the poultry house**	**According to you, does this measure implemented on the farms?**	**For a measure not always implemented on the farms**	
		**Could you explain the reasons?**	**Do you think that this measure would be an efficient biosecurity measure for the farms and why?**
Concrete surrounds around the house	☐ Always<br>☐ Sometimes<br>☐ Never<br>☐ Other frequency…………………………………<br>………………………………………………………..<br>☐ Unknown<br>☐ Not concerned<br><br>Details: ……………………………………………<br><br>…………………………………………………........	☐ Too expensive<br>☐ Take too much time<br>☐ Not enough trained<br>☐ Not enough advice<br>☐ No knowing advantages<br>☐ Not useful<br>☐ Not adapted to the farm<br>☐ Other reason ………………………………………..<br>………………………………………………………..<br>☐ Unknown<br><br>Details: ……………………………………………….<br><br>…………………………………………………………	☐ Yes<br>☐ Moderately<br>☐ No<br>☐ Other opinion…………………………………….<br>………………………………………………………..<br>☐ Unknown<br><br>Why? ………………………………………………...<br>………………………………………………………..
Hygiene lock with 2 separated zones (clean and dirty area)	☐ Always<br>☐ Sometimes<br>☐ Never<br>☐ Other frequency…………………………………<br>………………………………………………………..<br>☐ Unknown<br>☐ Not concerned<br><br>Details: ……………………………………………<br><br>…………………………………………………........	☐ Too expensive<br>☐ Take too much time<br>☐ Not enough trained<br>☐ Not enough advice<br>☐ No knowing advantages<br>☐ Not useful<br>☐ Not adapted to the farm<br>☐ Other reason ………………………………………..<br>………………………………………………………..<br>☐ Unknown<br><br>Details: ……………………………………………….<br><br>………………………………………………………….	☐ Yes<br>☐ Moderately<br>☐ No<br>☐ Other opinion……………………………………<br>…………………………………………………………<br>☐ Unknown<br><br>Why? …………………………………………………<br>………………………………………………………….

**Item I2 - Management of the material or litter in the poultry house**	**According to you, does this measure implemented on the farms?**	**For a measure not always implemented on the farms**	
		**Could you explain the reasons?**	**Do you think that this measure would be an efficient biosecurity measure for the farms and why?**
Recognizable separate material only for the poultry house	☐ Always<br>☐ Sometimes<br>☐ Never<br>☐ Other frequency…………………………………<br>………………………………………………………..<br>☐ Unknown<br>☐ Not concerned<br><br>Details: ……………………………………………<br><br>………………………………………………..........	☐ Too expensive<br>☐ Take too much time<br>☐ Not enough trained<br>☐ Not enough advice<br>☐ No knowing advantages<br>☐ Not useful<br>☐ Not adapted to the farm<br>☐ Other reason ………………………………………..<br>…………………………………………………………..<br>☐ Unknown<br>Details: ……………………………………………….<br><br>………………………………………………………….	☐ Yes<br>☐ Moderately<br>☐ No<br>☐ Other opinion………………………………………<br>…………………………………………………………<br>☐ Unknown<br><br>Why? …………………………………………………<br>………………………………………………………….
Protection of the litter (in a closed shed or other protection, from birds or vermin …)	☐ Always<br>☐ Sometimes<br>☐ Never<br>☐ Other frequency…………………………………<br>………………………………………………………..<br>☐ Unknown<br>☐ Not concerned<br><br>Details: ……………………………………………<br><br>………………………………………………..........	☐ Too expensive<br>☐ Take too much time<br>☐ Not enough trained<br>☐ Not enough advice<br>☐ No knowing advantages<br>☐ Not useful<br>☐ Not adapted to the farm<br>☐ Other reason ………………………………………..<br>…………………………………………………………..<br>☐ Unknown<br>Details: ……………………………………………….<br><br>………………………………………………………….	☐ Yes<br>☐ Moderately<br>☐ No<br>☐ Other opinion…………………………………….<br>………………………………………………………..<br>☐ Unknown<br><br>Why? ………………………………………………...<br>………………………………………………………..
**Item I3 - Cleaning and disinfection of the house and material**	**According to you, does this measure implemented on the farms?**	**For a measure not always implemented on the farms**	
		**Could you explain the reasons?**	**Do you think that this measure would be an efficient biosecurity measure for the farms and why?**
Cleaning and disinfection of the house between each flock	☐ Always<br>☐ Sometimes<br>☐ Never<br>☐ Other frequency…………………………………<br>………………………………………………………..<br>☐ Unknown<br>☐ Not concerned<br><br>Details: ……………………………………………<br><br>………………………………………………..........	☐ Too expensive<br>☐ Take too much time<br>☐ Not enough trained<br>☐ Not enough advice<br>☐ No knowing advantages<br>☐ Not useful<br>☐ Not adapted to the farm<br>☐ Other reason ………………………………………..<br>…………………………………………………………..<br>☐ Unknown<br><br>Details: ……………………………………………….<br><br>………………………………………………………….	☐ Yes<br>☐ Moderately<br>☐ No<br>☐ Other opinion………………………………………<br>…………………………………………………………<br>☐ Unknown<br><br>Why? …………………………………………………<br>………………………………………………………….

Cleaning and disinfection of the material between each flock (feeders, drinkers, nests, material for the management of eggs, …)	□ Always<br>□ Sometimes<br>□ Never<br>□ Other frequency……………………………………<br>……………………………………………………………..<br>□ Unknown<br>□ Not concerned<br><br>Details: ……………………………………………<br><br>…………………………………………………..........	□ Too expensive<br>□ Take too much time<br>□ Not enough trained<br>□ Not enough advice<br>□ No knowing advantages<br>□ Not useful<br>□ Not adapted to the farm<br>□ Other reason ……………………………………….<br>…………………………………………………………..<br>□ Unknown<br>Details: ………………………………………………	□ Yes<br>□ Moderately<br>□ No<br>□ Other opinion…………………………………….<br>……………………………………………………………..<br>□ Unknown<br><br>Why? ………………………………………………...<br>…………………………………………………………..
Cleaning and disinfection of the drinking water pipeline between each flock	□ Always<br>□ Sometimes<br>□ Never<br>□ Other frequency……………………………………<br>……………………………………………………………..<br>□ Unknown<br>□ Not concerned<br><br>Details: ……………………………………………<br><br>…………………………………………………..........	□ Too expensive<br>□ Take too much time<br>□ Not enough trained<br>□ Not enough advice<br>□ No knowing advantages<br>□ Not useful<br>□ Not adapted to the farm<br>□ Other reason ……………………………………….<br>…………………………………………………………..<br>□ Unknown<br>Details: ………………………………………………	□ Yes<br>□ Moderately<br>□ No<br>□ Other opinion……………………………………<br>…………………………………………………………<br>□ Unknown<br><br>Why? …………………………………………………<br>………………………………………………………….
Cleaning and disinfection of the feed silo between each flock	□ Always<br>□ Sometimes<br>□ Never<br>□ Other frequency……………………………………<br>……………………………………………………………..<br>□ Unknown<br>□ Not concerned<br><br>Details: ……………………………………………<br><br>…………………………………………………..........	□ Too expensive<br>□ Take too much time<br>□ Not enough trained<br>□ Not enough advice<br>□ No knowing advantages<br>□ Not useful<br>□ Not adapted to the farm<br>□ Other reason ……………………………………….<br>…………………………………………………………..<br>□ Unknown<br>Details: ………………………………………………	□ Yes<br>□ Moderately<br>□ No<br>□ Other opinion…………………………………….<br>……………………………………………………………..<br>□ Unknown<br><br>Why? ………………………………………………...<br>…………………………………………………………..
Bacterial autocontrol of the cleaning and disinfection of the house between each flock	□ Always<br>□ Sometimes<br>□ Never<br>□ Other frequency……………………………………<br>……………………………………………………………..<br>□ Unknown<br>□ Not concerned<br><br>Details: ……………………………………………<br><br>…………………………………………………..........	□ Too expensive<br>□ Take too much time<br>□ Not enough trained<br>□ Not enough advice<br>□ No knowing advantages<br>□ Not useful<br>□ Not adapted to the farm<br>□ Other reason ……………………………………….<br>…………………………………………………………..<br>□ Unknown<br><br>Details: ………………………………………………<br><br>………………………………………………………….	□ Yes<br>□ Moderately<br>□ No<br>□ Other opinion……………………………………<br>…………………………………………………………<br>□ Unknown<br><br>Why? …………………………………………………<br>………………………………………………………….

Period of the sanitary break > 15 days between each flock	☐ Always<br>☐ Sometimes<br>☐ Never<br>☐ Other frequency…………………………………<br>……………………………………………………..<br>☐ Unknown<br>☐ Not concerned<br><br>Details: ……………………………………………<br><br>………………………………………………..........	☐ Too expensive<br>☐ Take too much time<br>☐ Not enough trained<br>☐ Not enough advice<br>☐ No knowing advantages<br>☐ Not useful<br>☐ Not adapted to the farm<br>☐ Other reason ………………………………………..<br>…………………………………………………………..<br>☐ Unknown<br><br>Details: ………………………………………………<br><br>…………………………………………………………	☐ Yes<br>☐ Moderately<br>☐ No<br>☐ Other opinion…………………………………..<br>………………………………………………………..<br>☐ Unknown<br><br>Why? ………………………………………………...<br>………………………………………………………..
**Item I5 - Management of the poultry**	**According to you, does this measure implemented on the farms?**	**For a measure not always implemented on the farms**<br>**Could you explain the reasons?**	**Do you think that this measure would be an efficient biosecurity measure for the farms and why?**
Vaccination protocol of each poultry flock	☐ Always<br>☐ Sometimes<br>☐ Never<br>☐ Other frequency…………………………………<br>……………………………………………………..<br>☐ Unknown<br>☐ Not concerned<br><br>Details: ……………………………………………<br><br>………………………………………………..........	☐ Too expensive<br>☐ Take too much time<br>☐ Not enough trained<br>☐ Not enough advice<br>☐ No knowing advantages<br>☐ Not useful<br>☐ Not adapted to the farm<br>☐ Other reason ………………………………………..<br>…………………………………………………………..<br>☐ Unknown<br><br>Details: ……………………………………………….<br><br>………………………………………………………….	☐ Yes<br>☐ Moderately<br>☐ No<br>☐ Other opinion……………………………………..<br>…………………………………………………………<br>☐ Unknown<br><br>Why? ………………………………………………….<br>………………………………………………………….
Daily surveillance with clinical alert criteria (water and feed consummation, mortality, eggs production)	☐ Always<br>☐ Sometimes<br>☐ Never<br>☐ Other frequency…………………………………<br>……………………………………………………..<br>☐ Unknown<br>☐ Not concerned<br><br>Details: ……………………………………………<br><br>………………………………………………..........	☐ Too expensive<br>☐ Take too much time<br>☐ Not enough trained<br>☐ Not enough advice<br>☐ No knowing advantages<br>☐ Not useful<br>☐ Not adapted to the farm<br>☐ Other reason ………………………………………..<br>…………………………………………………………..<br>☐ Unknown<br><br>Details: ………………………………………………<br><br>…………………………………………………………	☐ Yes<br>☐ Moderately<br>☐ No<br>☐ Other opinion…………………………………..<br>………………………………………………………..<br>☐ Unknown<br><br>Why? ………………………………………………...<br>………………………………………………………..

Section B. Supplementary Biosecurity Item - Free range

Item I4 - Management of the free range area	According to you, does this measure implemented on the farms?	For a measure not always implemented on the farms<br>Could you explain the reasons?	Do you think that this measure would be an efficient biosecurity measure for the farms and why?
Free range area in the secured professional area without vehicle passage	☐ Always<br>☐ Sometimes<br>☐ Never<br>☐ Other frequency…………………………………<br>………………………………………………………..<br>☐ Unknown<br>☐ Not concerned<br><br>Details: ……………………………………………<br><br>………………………………………………..........	☐ Too expensive<br>☐ Take too much time<br>☐ Not enough trained<br>☐ Not enough advice<br>☐ No knowing advantages<br>☐ Not useful<br>☐ Not adapted to the farm<br>☐ Other reason ……………………………………….<br>………………………………………………………….<br>☐ Unknown<br>Details: ……………………………………………….<br><br>………………………………………………………….	☐ Yes<br>☐ Moderately<br>☐ No<br>☐ Other opinion……………………………………<br>………………………………………………………….<br>☐ Unknown<br><br>Why? ……………………………………………….<br>………………………………………………………….
Free range area away from a waterway	☐ Always<br>☐ Sometimes<br>☐ Never<br>☐ Other frequency…………………………………<br>………………………………………………………..<br>☐ Unknown<br>☐ Not concerned<br><br>Details: ……………………………………………<br><br>………………………………………………..........	☐ Too expensive<br>☐ Take too much time<br>☐ Not enough trained<br>☐ Not enough advice<br>☐ No knowing advantages<br>☐ Not useful<br>☐ Not adapted to the farm<br>☐ Other reason ……………………………………….<br>………………………………………………………….<br>☐ Unknown<br>Details: ……………………………………………….<br><br>………………………………………………………….	☐ Yes<br>☐ Moderately<br>☐ No<br>☐ Other opinion…………………………………..<br>…………………………………………………………..<br>☐ Unknown<br><br>Why? ……………………………………………...<br>………………………………………………………...
Free range area closed	☐ Always<br>☐ Sometimes<br>☐ Never<br>☐ Other frequency…………………………………<br>………………………………………………………..<br>☐ Unknown<br>☐ Not concerned<br><br>Details: ……………………………………………<br><br>………………………………………………..........	☐ Too expensive<br>☐ Take too much time<br>☐ Not enough trained<br>☐ Not enough advice<br>☐ No knowing advantages<br>☐ Not useful<br>☐ Not adapted to the farm<br>☐ Other reason ……………………………………….<br>………………………………………………………….<br>☐ Unknown<br>Details: ……………………………………………….<br><br>………………………………………………………….	☐ Yes<br>☐ Moderately<br>☐ No<br>☐ Other opinion……………………………………<br>………………………………………………………….<br>☐ Unknown<br><br>Why? ……………………………………………….<br>………………………………………………………….

Concrete exit area to avoid stagnant water in front of the house	□ Always<br>□ Sometimes<br>□ Never<br>□ Other frequency…………………………………<br>…………………………………………………………<br>□ Unknown<br>□ Not concerned<br><br>Details: ……………………………………………<br><br>……………………………………………………...	□ Too expensive<br>□ Take too much time<br>□ Not enough trained<br>□ Not enough advice<br>□ No knowing advantages<br>□ Not useful<br>□ Not adapted to the farm<br>□ Other reason ………………………………………..<br>…………………………………………………………..<br>□ Unknown<br>Details: ………………………………………………	□ Yes<br>□ Moderately<br>□ No<br>□ Other opinion……………………………………<br>……………………………………………………………..<br>□ Unknown<br><br>Why? ……………………………………………...<br>…………………………………………………………..
No stagnant water on the free range area	□ Always<br>□ Sometimes<br>□ Never<br>□ Other frequency…………………………………<br>…………………………………………………………<br>□ Unknown<br>□ Not concerned<br><br>Details: ……………………………………………<br><br>……………………………………………………...	□ Too expensive<br>□ Take too much time<br>□ Not enough trained<br>□ Not enough advice<br>□ No knowing advantages<br>□ Not useful<br>□ Not adapted to the farm<br>□ Other reason ………………………………………..<br>…………………………………………………………..<br>□ Unknown<br>Details: ………………………………………………	□ Yes<br>□ Moderately<br>□ No<br>□ Other opinion………………………………………<br>……………………………………………………………<br>□ Unknown<br><br>Why? ………………………………………………<br>……………………………………………………………
Protective net on the free range area (with anti-roost system)	□ Always<br>□ Sometimes<br>□ Never<br>□ Other frequency…………………………………<br>…………………………………………………………<br>□ Unknown<br>□ Not concerned<br><br>Details: ……………………………………………<br><br>……………………………………………………...	□ Too expensive<br>□ Take too much time<br>□ Not enough trained<br>□ Not enough advice<br>□ No knowing advantages<br>□ Not useful<br>□ Not adapted to the farm<br>□ Other reason ………………………………………..<br>…………………………………………………………..<br>□ Unknown<br>Details: ………………………………………………	□ Yes<br>□ Moderately<br>□ No<br>□ Other opinion……………………………………<br>……………………………………………………………..<br>□ Unknown<br><br>Why? ……………………………………………...<br>…………………………………………………………..
No food and drinking water outside the house	□ Always<br>□ Sometimes<br>□ Never<br>□ Other frequency…………………………………<br>…………………………………………………………<br>□ Unknown<br>□ Not concerned<br><br>Details: ……………………………………………<br><br>……………………………………………………...	□ Too expensive<br>□ Take too much time<br>□ Not enough trained<br>□ Not enough advice<br>□ No knowing advantages<br>□ Not useful<br>□ Not adapted to the farm<br>□ Other reason ………………………………………..<br>…………………………………………………………..<br>□ Unknown<br><br>Details: ………………………………………………<br><br>……………………………………………………………	□ Yes<br>□ Moderately<br>□ No<br>□ Other opinion………………………………………<br>……………………………………………………………<br>□ Unknown<br><br>Why? ………………………………………………<br>……………………………………………………………

Maintenance and disinfection of the free range area	☐ Always<br>☐ Sometimes<br>☐ Never<br>☐ Other frequency…………………………………<br>…………………………………………………….<br>☐ Unknown<br>☐ Not concerned<br><br>Details: ……………………………………………<br>……………………………………………….........	☐ Too expensive<br>☐ Take too much time<br>☐ Not enough trained<br>☐ Not enough advice<br>☐ No knowing advantages<br>☐ Not useful<br>☐ Not adapted to the farm<br>☐ Other reason ……………………………………….<br>…………………………………………………………..<br>☐ Unknown<br><br>Details: ……………………………………………….<br>………………………………………………………….	☐ Yes<br>☐ Moderately<br>☐ No<br>☐ Other opinion…………………………………….<br>…………………………………………………………..<br>☐ Unknown<br><br>Why? ………………………………………………...<br>………………………………………………………..
Period of sanitary break of the free range area	☐ Always<br>☐ Sometimes<br>☐ Never<br>☐ Other frequency…………………………………<br>…………………………………………………….<br>☐ Unknown<br>☐ Not concerned<br><br>Details: ……………………………………………<br>……………………………………………….........	☐ Too expensive<br>☐ Take too much time<br>☐ Not enough trained<br>☐ Not enough advice<br>☐ No knowing advantages<br>☐ Not useful<br>☐ Not adapted to the farm<br>☐ Other reason ……………………………………….<br>…………………………………………………………..<br>☐ Unknown<br><br>Details: ……………………………………………….<br>………………………………………………………….	☐ Yes<br>☐ Moderately<br>☐ No<br>☐ Other opinion…………………………………….<br>…………………………………………………………..<br>☐ Unknown<br><br>Why? ………………………………………………...<br>………………………………………………………..

Section C.Supplementary Biosecurity Item - Breeder

**Item I5 - Management of the hatching eggs**	**According to you, does this measure implemented on the farms?**	**For a measure not always implemented on the farms**	
		**Could you explain the reasons?**	**Do you think that this measure would be an efficient biosecurity measure for the farms and why?**
Automatic hatching eggs collection system	☐ Always<br>☐ Sometimes<br>☐ Never<br>☐ Other frequency………………………………….<br>……………………………………………………….<br>☐ Unknown<br>☐ Not concerned<br><br>Details: ……………………………………………<br>………………………………………………..........	☐ Too expensive<br>☐ Take too much time<br>☐ Not enough trained<br>☐ Not enough advice<br>☐ No knowing advantages<br>☐ Not useful<br>☐ Not adapted to the farm<br>☐ Other reason ………………………………………..<br>…………………………………………………………..<br>☐ Unknown<br>Details: ……………………………………………….<br>…………………………………………………………	☐ Yes<br>☐ Moderately<br>☐ No<br>☐ Other opinion…………………………………….<br>……………………………………………………….<br>☐ Unknown<br><br>Why? ………………………………………………...<br>………………………………………………………..
Traceability of the hatching eggs (breeder flock, laying date …)	☐ Always<br>☐ Sometimes<br>☐ Never<br>☐ Other frequency………………………………….<br>……………………………………………………….<br>☐ Unknown<br>☐ Not concerned<br><br>Details: ……………………………………………<br>………………………………………………..........	☐ Too expensive<br>☐ Take too much time<br>☐ Not enough trained<br>☐ Not enough advice<br>☐ No knowing advantages<br>☐ Not useful<br>☐ Not adapted to the farm<br>☐ Other reason ………………………………………..<br>…………………………………………………………..<br>☐ Unknown<br><br>Details: ……………………………………………….<br>…………………………………………………………	☐ Yes<br>☐ Moderately<br>☐ No<br>☐ Other opinion…………………………………….<br>……………………………………………………….<br>☐ Unknown<br><br>Why? ………………………………………………...<br>………………………………………………………..
Hatching eggs disinfection at the farm	☐ Always<br>☐ Sometimes<br>☐ Never<br>☐ Other frequency………………………………….<br>……………………………………………………….<br>☐ Unknown<br>☐ Not concerned<br><br>Details: ……………………………………………<br>………………………………………………..........	☐ Too expensive<br>☐ Take too much time<br>☐ Not enough trained<br>☐ Not enough advice<br>☐ No knowing advantages<br>☐ Not useful<br>☐ Not adapted to the farm<br>☐ Other reason ………………………………………..<br>…………………………………………………………..<br>☐ Unknown<br>Details: ……………………………………………….<br>…………………………………………………………	☐ Yes<br>☐ Moderately<br>☐ No<br>☐ Other opinion…………………………………….<br>……………………………………………………….<br>☐ Unknown<br><br>Why? ………………………………………………...<br>………………………………………………………..

Specific hatching eggs sorting and storage rooms	☐ Always<br>☐ Sometimes<br>☐ Never<br>☐ Other frequency…………………………………<br>………………………………………………………..<br>☐ Unknown<br>☐ Not concerned<br><br>Details: ……………………………………………<br><br>…………………………………………………..........	☐ Too expensive<br>☐ Take too much time<br>☐ Not enough trained<br>☐ Not enough advice<br>☐ No knowing advantages<br>☐ Not useful<br>☐ Not adapted to the farm<br>☐ Other reason ………………………………………..<br>……………………………………………………………..<br>☐ Unknown<br>Details: ………………………………………………	☐ Yes<br>☐ Moderately<br>☐ No<br>☐ Other opinion…………………………………….<br>………………………………………………………….<br>☐ Unknown<br><br>Why? …………………………………………………...<br>……………………………………………………….
Specific entrance for the collection of the hatching eggs by the driver	☐ Always<br>☐ Sometimes<br>☐ Never<br>☐ Other frequency…………………………………<br>………………………………………………………..<br>☐ Unknown<br>☐ Not concerned<br><br>Details: ……………………………………………<br><br>…………………………………………………..........	☐ Too expensive<br>☐ Take too much time<br>☐ Not enough trained<br>☐ Not enough advice<br>☐ No knowing advantages<br>☐ Not useful<br>☐ Not adapted to the farm<br>☐ Other reason ………………………………………..<br>……………………………………………………………..<br>☐ Unknown<br>Details: ………………………………………………	☐ Yes<br>☐ Moderately<br>☐ No<br>☐ Other opinion…………………………………….<br>………………………………………………………….<br>☐ Unknown<br><br>Why? …………………………………………………...<br>……………………………………………………….
If the driver enters in the storage rooms for the collection of eggs: specific clothes and shoes	☐ Always<br>☐ Sometimes<br>☐ Never<br>☐ Other frequency…………………………………<br>………………………………………………………..<br>☐ Unknown<br>☐ Not concerned<br><br>Details: ……………………………………………<br><br>…………………………………………………..........	☐ Too expensive<br>☐ Take too much time<br>☐ Not enough trained<br>☐ Not enough advice<br>☐ No knowing advantages<br>☐ Not useful<br>☐ Not adapted to the farm<br>☐ Other reason ………………………………………..<br>……………………………………………………………..<br>☐ Unknown<br>Details: ………………………………………………	☐ Yes<br>☐ Moderately<br>☐ No<br>☐ Other opinion…………………………………….<br>………………………………………………………….<br>☐ Unknown<br><br>Why? …………………………………………………...<br>……………………………………………………….
Cleaning and disinfection of the storage room after each collection	☐ Always<br>☐ Sometimes<br>☐ Never<br>☐ Other frequency…………………………………<br>………………………………………………………..<br>☐ Unknown<br>☐ Not concerned<br><br>Details: ……………………………………………<br><br>…………………………………………………..........	☐ Too expensive<br>☐ Take too much time<br>☐ Not enough trained<br>☐ Not enough advice<br>☐ No knowing advantages<br>☐ Not useful<br>☐ Not adapted to the farm<br>☐ Other reason ………………………………………..<br>……………………………………………………………..<br>☐ Unknown<br><br>Details: ………………………………………………<br><br>………………………………………………………	☐ Yes<br>☐ Moderately<br>☐ No<br>☐ Other opinion…………………………………….<br>………………………………………………………….<br>☐ Unknown<br><br>Why? …………………………………………………...<br>……………………………………………………….

Different personal between the poultry room and the eggs sorting and storage rooms	□ Always<br>□ Sometimes<br>□ Never<br>□ Other frequency…………………………………<br>……………………………………………………..<br>□ Unknown<br>□ Not concerned<br><br>Details: ……………………………………………<br><br>……………………………………………….........	□ Too expensive<br>□ Take too much time<br>□ Not enough trained<br>□ Not enough advice<br>□ No knowing advantages<br>□ Not useful<br>□ Not adapted to the farm<br>□ Other reason ………………………………………..<br>….…………………………………………………………..<br>□ Unknown<br><br>Details: ……………………………………………….<br><br>……………………………………………………………	□ Yes<br>□ Moderately<br>□ No<br>□ Other opinion……………………………………<br>……………………………………………………..<br>□ Unknown<br><br>Why? ………………………………………………...<br>……………………………………………………….

Section D.Supplementary Biosecurity Item - Layers

Item I5 - Management of the laying eggs	According to you, does this measure implemented on the farms?	For a measure not always implemented on the farms<br>Could you explain the reasons?	Do you think that this measure would be an efficient biosecurity measure for the farms and why?
Automatic laying eggs collection system	□ Always<br>□ Sometimes<br>□ Never<br>□ Other frequency…………………………………<br>……………………………………………………….<br>□ Unknown<br>□ Not concerned<br><br>Details: ……………………………………………<br><br>………………………………………………..........	□ Too expensive<br>□ Take too much time<br>□ Not enough trained<br>□ Not enough advice<br>□ No knowing advantages<br>□ Not useful<br>□ Not adapted to the farm<br>□ Other reason ………………………………………..<br>…………………………………………………………..<br>□ Unknown<br><br>Details: ………………………………………………	□ Yes<br>□ Moderately<br>□ No<br>□ Other opinion……………………………………<br>……………………………………………………….<br>□ Unknown<br><br>Why? ………………………………………………...<br>………………………………………………………..
Traceability of the laying eggs (layers flock, laying date …)	□ Always<br>□ Sometimes<br>□ Never<br>□ Other frequency…………………………………<br>……………………………………………………….<br>□ Unknown<br>□ Not concerned<br><br>Details: ……………………………………………<br><br>………………………………………………..........	□ Too expensive<br>□ Take too much time<br>□ Not enough trained<br>□ Not enough advice<br>□ No knowing advantages<br>□ Not useful<br>□ Not adapted to the farm<br>□ Other reason ………………………………………..<br>…………………………………………………………..<br>□ Unknown<br><br>Details: ………………………………………………<br><br>…………………………………………………………	□ Yes<br>□ Moderately<br>□ No<br>□ Other opinion……………………………………<br>……………………………………………………….<br>□ Unknown<br><br>Why? ………………………………………………...<br>………………………………………………………..
Specific laying eggs sorting and storage rooms	□ Always<br>□ Sometimes<br>□ Never<br>□ Other frequency…………………………………<br>……………………………………………………….<br>□ Unknown<br>□ Not concerned<br><br>Details: ……………………………………………<br><br>………………………………………………..........	□ Too expensive<br>□ Take too much time<br>□ Not enough trained<br>□ Not enough advice<br>□ No knowing advantages<br>□ Not useful<br>□ Not adapted to the farm<br>□ Other reason ………………………………………..<br>…………………………………………………………..<br>□ Unknown<br><br>Details: ………………………………………………	□ Yes<br>□ Moderately<br>□ No<br>□ Other opinion……………………………………<br>……………………………………………………….<br>□ Unknown<br><br>Why? ………………………………………………...<br>………………………………………………………..

Specific entrance for the collection of the eggs by the driver	☐ Always<br>☐ Sometimes<br>☐ Never<br>☐ Other frequency…………………………………<br>……………………………………………………….<br>☐ Unknown<br>☐ Not concerned<br><br>Details: ……………………………………………<br><br>………………………………………………….........	☐ Too expensive<br>☐ Take too much time<br>☐ Not enough trained<br>☐ Not enough advice<br>☐ No knowing advantages<br>☐ Not useful<br>☐ Not adapted to the farm<br>☐ Other reason ………………………………………..<br>……………………………………………………………..<br>☐ Unknown<br>Details: ………………………………………………	☐ Yes<br>☐ Moderately<br>☐ No<br>☐ Other opinion……………………………………<br>…………………………………………………………..<br>☐ Unknown<br><br>Why? ………………………………………………...<br>…………………………………………………………..
If the driver enters in the storage rooms for the collection of eggs: specific clothes and shoes	☐ Always<br>☐ Sometimes<br>☐ Never<br>☐ Other frequency…………………………………<br>……………………………………………………….<br>☐ Unknown<br>☐ Not concerned<br><br>Details: ……………………………………………<br><br>………………………………………………….........	☐ Too expensive<br>☐ Take too much time<br>☐ Not enough trained<br>☐ Not enough advice<br>☐ No knowing advantages<br>☐ Not useful<br>☐ Not adapted to the farm<br>☐ Other reason ………………………………………..<br>……………………………………………………………..<br>☐ Unknown<br><br>Details: ………………………………………………	☐ Yes<br>☐ Moderately<br>☐ No<br>☐ Other opinion……………………………………<br>…………………………………………………………..<br>☐ Unknown<br><br>Why? ………………………………………………...<br>…………………………………………………………..
Cleaning and disinfection of the storage room after each collection	☐ Always<br>☐ Sometimes<br>☐ Never<br>☐ Other frequency…………………………………<br>……………………………………………………….<br>☐ Unknown<br>☐ Not concerned<br><br>Details: ……………………………………………<br><br>………………………………………………….........	☐ Too expensive<br>☐ Take too much time<br>☐ Not enough trained<br>☐ Not enough advice<br>☐ No knowing advantages<br>☐ Not useful<br>☐ Not adapted to the farm<br>☐ Other reason ………………………………………..<br>……………………………………………………………..<br>☐ Unknown<br>Details: ………………………………………………	☐ Yes<br>☐ Moderately<br>☐ No<br>☐ Other opinion……………………………………<br>…………………………………………………………..<br>☐ Unknown<br><br>Why? ………………………………………………...<br>…………………………………………………………..
Different personal between the poultry room and the eggs sorting and storage room	☐ Always<br>☐ Sometimes<br>☐ Never<br>☐ Other frequency…………………………………<br>……………………………………………………….<br>☐ Unknown<br>☐ Not concerned<br><br>Details: ……………………………………………<br><br>………………………………………………….........	☐ Too expensive<br>☐ Take too much time<br>☐ Not enough trained<br>☐ Not enough advice<br>☐ No knowing advantages<br>☐ Not useful<br>☐ Not adapted to the farm<br>☐ Other reason ………………………………………..<br>……………………………………………………………..<br>☐ Unknown<br>Details: ………………………………………………	☐ Yes<br>☐ Moderately<br>☐ No<br>☐ Other opinion……………………………………<br>…………………………………………………………..<br>☐ Unknown<br><br>Why? ………………………………………………...<br>…………………………………………………………..

Section E. Other Biosecurity measures not always implemented on the farms – All the poultry

**Are there other biosecurity measures that you advice but that are not always implemented on the farms? ☐ Yes ☐ No**		
	**Could you explain the reasons?**	**Why do you think that this measure would be an efficient biosecurity measure for the farms?**
**Measure 1** ……………………………………………...<br>……………………………………………………………<br>……………………………………………………………	☐ Too expensive<br>☐ Take too much time<br>☐ Not enough trained<br>☐ Not enough advice<br>☐ No knowing advantages<br>☐ Not useful<br>☐ Not adapted to the farm<br>☐ Unknown<br>☐ Other reason ……………………………………………………<br>……………………………………………………………………………<br><br>Details : ………………………………………………………………<br>……………………………………………………………………………	……………………………………………………………………………<br>……………………………………………………………………………<br>……………………………………………………………………………<br>……………………………………………………………………………<br>……………………………………………………………………………<br>……………………………………………………………………………<br>……………………………………………………………………………<br>……………………………………………………………………………<br>……………………………………………………………………………<br>…………………………………………………………………………….
**Measure 2** ……………………………………………...<br>……………………………………………………………<br>……………………………………………………………	☐ Too expensive<br>☐ Take too much time<br>☐ Not enough trained<br>☐ Not enough advice<br>☐ No knowing advantages<br>☐ Not useful<br>☐ Not adapted to the farm<br>☐ Unknown<br>☐ Other reason ……………………………………………………<br>……………………………………………………………………………<br><br>Details : ……………………………………………………………….<br>……………………………………………………………………………..	……………………………………………………………………………<br>……………………………………………………………………………<br>……………………………………………………………………………<br>……………………………………………………………………………<br>……………………………………………………………………………<br>……………………………………………………………………………<br>……………………………………………………………………………<br>……………………………………………………………………………<br>……………………………………………………………………………<br>…………………………………………………………………………….
**Measure 3** ……………………………………………...<br>……………………………………………………………<br>……………………………………………………………	☐ Too expensive<br>☐ Take too much time<br>☐ Not enough trained<br>☐ Not enough advice<br>☐ No knowing advantages<br>☐ Not useful<br>☐ Not adapted to the farm<br>☐ Unknown<br>☐ Other reason ……………………………………………………<br>……………………………………………………………………………<br>Details : ………………………………………………………………<br>……………………………………………………………………………	……………………………………………………………………………<br>……………………………………………………………………………<br>……………………………………………………………………………<br>……………………………………………………………………………<br>……………………………………………………………………………<br>……………………………………………………………………………<br>……………………………………………………………………………<br>……………………………………………………………………………<br>……………………………………………………………………………<br>…………………………………………………………………………….

**Measure 4** ……………………………………………..<br>…………………………………………………………<br>…………………………………………………………	☐ Too expensive<br>☐ Take too much time<br>☐ Not enough trained<br>☐ Not enough advice<br>☐ No knowing advantages<br>☐ Not useful<br>☐ Not adapted to the farm<br>☐ Unknown<br>☐ Other reason ……………………………………………………<br>……………………………………………………………………<br>Details : …………………………………………………………<br>……………………………………………………………………	……………………………………………………………………<br>……………………………………………………………………<br>……………………………………………………………………<br>……………………………………………………………………<br>……………………………………………………………………<br>……………………………………………………………………<br>……………………………………………………………………<br>……………………………………………………………………<br>……………………………………………………………………<br>……………………………………………………………………
**Measure 5** ……………………………………………..<br>…………………………………………………………<br>…………………………………………………………	☐ Too expensive<br>☐ Take too much time<br>☐ Not enough trained<br>☐ Not enough advice<br>☐ No knowing advantages<br>☐ Not useful<br>☐ Not adapted to the farm<br>☐ Unknown<br>☐ Other reason ……………………………………………………<br>……………………………………………………………………<br>Details : …………………………………………………………<br>……………………………………………………………………..	……………………………………………………………………<br>……………………………………………………………………<br>……………………………………………………………………<br>……………………………………………………………………<br>……………………………………………………………………<br>……………………………………………………………………<br>……………………………………………………………………<br>……………………………………………………………………<br>……………………………………………………………………<br>……………………………………………………………………
**Measure 6** ……………………………………………..<br>…………………………………………………………<br>…………………………………………………………	☐ Too expensive<br>☐ Take too much time<br>☐ Not enough trained<br>☐ Not enough advice<br>☐ No knowing advantages<br>☐ Not useful<br>☐ Not adapted to the farm<br>☐ Unknown<br>☐ Other reason ……………………………………………………<br>……………………………………………………………………<br>Details : …………………………………………………………<br>……………………………………………………………………	……………………………………………………………………<br>……………………………………………………………………<br>……………………………………………………………………<br>……………………………………………………………………<br>……………………………………………………………………<br>……………………………………………………………………<br>……………………………………………………………………<br>……………………………………………………………………<br>……………………………………………………………………<br>……………………………………………………………………

Section F. Successful and Required Supporting measures – All the poultry

Item S1 - Biosecurity trainings	Which successful supporting measures have already helped the farmers to implement biosecurity measures on their farm?	Which required supporting measures the farmers would need to improve the implementation of the biosecurity measures on their farm?	In your opinion, why do you think that these Supporting measures that you have mentioned here (successful and required) are effective to improve biosecurity on the farms?	For the required Supporting measures not implemented on the farms that you have mentioned here:	
				Could you explain the reasons?	What solution or what would it need to implement these supporting measures on the farms?
exposure visit at well-organized farm/company/field trip	☐<br>Details<br>………………………………<br>………………………………	☐<br>Details<br>…………………………………<br>…………………………………	…………………………………<br>…………………………………<br>…………………………………<br>…………………………………	…………………………………<br>…………………………………<br>…………………………………<br>…………………………………	…………………………………<br>…………………………………<br>…………………………………<br>…………………………………
group discussion	☐<br>Details<br>………………………………<br>………………………………	☐<br>Details<br>…………………………………<br>…………………………………	…………………………………<br>…………………………………<br>…………………………………<br>…………………………………	…………………………………<br>…………………………………<br>…………………………………<br>…………………………………	…………………………………<br>…………………………………<br>…………………………………<br>…………………………………
live workshops	☐<br>Details<br>………………………………<br>………………………………	☐<br>Details<br>…………………………………<br>…………………………………	…………………………………<br>…………………………………<br>…………………………………<br>…………………………………	…………………………………<br>…………………………………<br>…………………………………<br>…………………………………	…………………………………<br>…………………………………<br>…………………………………<br>…………………………………
videos	☐<br>Details<br>………………………………<br>…………………………………	☐<br>Details<br>…………………………………<br>…………………………………	…………………………………<br>…………………………………<br>…………………………………	…………………………………<br>…………………………………<br>…………………………………	…………………………………<br>…………………………………<br>…………………………………

webinars	☐<br>Details<br>………………………………<br>………………………………	☐<br>Details<br>…………………………………<br>…………………………………	…………………………………<br>…………………………………<br>…………………………………<br>…………………………………	…………………………………<br>…………………………………<br>…………………………………<br>…………………………………	…………………………………<br>…………………………………<br>…………………………………<br>…………………………………
educational modules	☐<br>Details<br>………………………………<br>………………………………	☐<br>Details<br>…………………………………<br>…………………………………	…………………………………<br>…………………………………<br>…………………………………<br>…………………………………	…………………………………<br>…………………………………<br>…………………………………<br>…………………………………	…………………………………<br>…………………………………<br>…………………………………<br>…………………………………
Other measure 1 in this item<br>………………………<br>………………………	☐<br>Details<br>………………………………<br>………………………………	☐<br>Details<br>…………………………………<br>…………………………………	…………………………………<br>…………………………………<br>…………………………………<br>…………………………………	…………………………………<br>…………………………………<br>…………………………………<br>…………………………………	…………………………………<br>…………………………………<br>…………………………………<br>…………………………………
Other measure 2 in this item<br>………………………<br>………………………	☐<br>Details<br>………………………………<br>………………………………	☐<br>Details<br>…………………………………<br>…………………………………	…………………………………<br>…………………………………<br>…………………………………<br>…………………………………	…………………………………<br>…………………………………<br>…………………………………<br>…………………………………	…………………………………<br>…………………………………<br>…………………………………<br>…………………………………
Other measure 3 in this item<br>………………………<br>………………………	☐<br>Details<br>………………………………<br>………………………………	☐<br>Details<br>…………………………………<br>…………………………………	…………………………………<br>…………………………………<br>…………………………………<br>…………………………………	…………………………………<br>…………………………………<br>…………………………………<br>…………………………………	…………………………………<br>…………………………………<br>…………………………………<br>…………………………………

Item S2 - Conducting information campaigns promoting biosecurity	Which successful supporting measures have already helped the farmers to implement biosecurity measures on their farm?	Which required supporting measures the farmers would need to improve the implementation of the biosecurity measures on their farm?	In your opinion, why do you think that these Supporting measures that you have mentioned here (successful and required) are effective to improve biosecurity on the farms?	For the required Supporting measures not implemented on the farms that you have mentioned here:	
				Could you explain the reasons?	What solution or what would it need to implement these supporting measures on the farms?
conferences/webinars advisiors meeting	☐ Details ………………………………… …………………………………	☐ Details …………………………………… ……………………………………	…………………………………… …………………………………… …………………………………… ……………………………………	…………………………………… …………………………………… …………………………………… ……………………………………	…………………………………… …………………………………… …………………………………… ……………………………………
Leaflets/banners/posters	☐ Details ………………………………… …………………………………	☐ Details …………………………………… ……………………………………	…………………………………… …………………………………… …………………………………… ……………………………………	…………………………………… …………………………………… …………………………………… ……………………………………	…………………………………… …………………………………… …………………………………… ……………………………………
media (TV and web: Youtube etc)	☐ Details ………………………………… …………………………………	☐ Details …………………………………… ……………………………………	…………………………………… …………………………………… …………………………………… ……………………………………	…………………………………… …………………………………… …………………………………… ……………………………………	…………………………………… …………………………………… …………………………………… ……………………………………
social media groups (Facebook, LinkedIn, etc)	☐ Details ………………………………… …………………………………	☐ Details …………………………………… ……………………………………	…………………………………… …………………………………… …………………………………… ……………………………………	…………………………………… …………………………………… …………………………………… ……………………………………	…………………………………… …………………………………… …………………………………… ……………………………………
Gadgets: (lines, pencils, key rings, cups, bags etc.)	☐ Details ………………………………… …………………………………	☐ Details …………………………………… ……………………………………	…………………………………… …………………………………… …………………………………… ……………………………………	…………………………………… …………………………………… …………………………………… ……………………………………	…………………………………… …………………………………… …………………………………… ……………………………………

Other measure 1 in this item<br>………………………<br>……………………...	☐<br>Details<br>………………………………<br>………………………………	☐<br>Details<br>…………………………………<br>…………………………………	…………………………………<br>…………………………………<br>…………………………………<br>…………………………………	…………………………………<br>…………………………………<br>…………………………………<br>…………………………………	…………………………………<br>…………………………………<br>…………………………………<br>…………………………………
Other measure 2 in this item<br>………………………<br>………………………	☐<br>Details<br>………………………………<br>………………………………	☐<br>Details<br>…………………………………<br>…………………………………	…………………………………<br>…………………………………<br>…………………………………<br>…………………………………	…………………………………<br>…………………………………<br>…………………………………<br>…………………………………	…………………………………<br>…………………………………<br>…………………………………<br>…………………………………
Other measure 3 in this item<br>………………………<br>………………………	☐<br>Details<br>………………………………<br>………………………………	☐<br>Details<br>…………………………………<br>…………………………………	…………………………………<br>…………………………………<br>…………………………………<br>…………………………………	…………………………………<br>…………………………………<br>…………………………………<br>…………………………………	…………………………………<br>…………………………………<br>…………………………………<br>…………………………………
**Item S3 - Educational material**	**Which successful supporting measures have already helped the farmers to implement biosecurity measures on their farm?**	**Which required supporting measures the farmers would need to improve the implementation of the biosecurity measures on their farm?**	**In your opinion, why do you think that these Supporting measures that you have mentioned here (successful and required) are effective to improve biosecurity on the farms?**	**For the required Supporting measures not implemented on the farms that you have mentioned here:**	
				**Could you explain the reasons?**	**What solution or what would it need to implement these supporting measures on the farms?**
Books/guides/manuals/ research papers/journals/ farming press	☐<br>Details<br>………………………………<br>………………………………	☐<br>Details<br>…………………………………<br>…………………………………	…………………………………<br>…………………………………<br>…………………………………<br>…………………………………	…………………………………<br>…………………………………<br>…………………………………<br>…………………………………	…………………………………<br>…………………………………<br>…………………………………<br>…………………………………
Posters/banners/newsletters /leaflets	☐<br>Details<br>………………………………<br>………………………………	☐<br>Details<br>…………………………………<br>…………………………………	…………………………………<br>…………………………………<br>…………………………………<br>…………………………………	…………………………………<br>…………………………………<br>…………………………………<br>…………………………………	…………………………………<br>…………………………………<br>…………………………………<br>…………………………………

Media: TV and web (YouTube etc.)	☐ Details ………………………………… …………………………………	☐ Details ………………………………… …………………………………	………………………………… ………………………………… ………………………………… …………………………………	………………………………… ………………………………… ………………………………… …………………………………	………………………………… ………………………………… ………………………………… …………………………………
social media groups (Facebook, LinkedIn, etc)	☐ Details ………………………………… …………………………………	☐ Details ………………………………… …………………………………	………………………………… ………………………………… ………………………………… …………………………………	………………………………… ………………………………… ………………………………… …………………………………	………………………………… ………………………………… ………………………………… …………………………………
Other measure 1 in this item ……………………… ………………………	☐ Details ………………………………… …………………………………	☐ Details ………………………………… …………………………………	………………………………… ………………………………… ………………………………… …………………………………	………………………………… ………………………………… ………………………………… …………………………………	………………………………… ………………………………… ………………………………… …………………………………
Other measure 2 in this item ……………………… ………………………	☐ Details ………………………………… …………………………………	☐ Details ………………………………… …………………………………	………………………………… ………………………………… ………………………………… …………………………………	………………………………… ………………………………… ………………………………… …………………………………	………………………………… ………………………………… ………………………………… …………………………………
Other measure 3 in this item ……………………… ………………………	☐ Details ………………………………… …………………………………	☐ Details ………………………………… …………………………………	………………………………… ………………………………… ………………………………… …………………………………	………………………………… ………………………………… ………………………………… …………………………………	………………………………… ………………………………… ………………………………… …………………………………

**Item S4 - Biosecurity checks (audits)**	**Which successful supporting measures have already helped the farmers to implement biosecurity measures on their farm?**	**Which required supporting measures the farmers would need to improve the implementation of the biosecurity measures on their farm?**	**In your opinion, why do you think that these Supporting measures that you have mentioned here (successful and required) are effective to improve biosecurity on the farms?**	**For the required Supporting measures not implemented on the farms that you have mentioned here:**	
				**Could you explain the reasons?**	**What solution or what would it need to implement these supporting measures on the farms?**
by government	☐<br>Details<br>……………………………<br>……………………………	☐<br>Details<br>……………………………<br>……………………………	……………………………<br>……………………………<br>……………………………<br>……………………………	……………………………<br>……………………………<br>……………………………<br>……………………………	……………………………<br>……………………………<br>……………………………<br>……………………………
by stakeholders (local integration companies etc.)	☐<br>Details<br>……………………………<br>……………………………	☐<br>Details<br>……………………………<br>……………………………	……………………………<br>……………………………<br>……………………………<br>……………………………	……………………………<br>……………………………<br>……………………………<br>……………………………	……………………………<br>……………………………<br>……………………………<br>……………………………
Other measure 1 in this item<br>………………………<br>………………………..	☐<br>Details<br>……………………………<br>……………………………	☐<br>Details<br>……………………………<br>……………………………	……………………………<br>……………………………<br>……………………………<br>……………………………	……………………………<br>……………………………<br>……………………………<br>……………………………	……………………………<br>……………………………<br>……………………………<br>……………………………
Other measure 2 in this item<br>………………………<br>………………………	☐<br>Details<br>……………………………<br>……………………………	☐<br>Details<br>……………………………<br>……………………………	……………………………<br>……………………………<br>……………………………<br>……………………………	……………………………<br>……………………………<br>……………………………<br>……………………………	……………………………<br>……………………………<br>……………………………<br>……………………………
Other measure 3 in this item<br>………………………<br>………………………	☐<br>Details<br>……………………………<br>……………………………	☐<br>Details<br>……………………………<br>……………………………	……………………………<br>……………………………<br>……………………………<br>……………………………	……………………………<br>……………………………<br>……………………………<br>……………………………	……………………………<br>……………………………<br>……………………………<br>……………………………

**Item S5 - Regulations set up supporting biosecurity implementation**	**Which successful supporting measures have already helped the farmers to implement biosecurity measures on their farm?**	**Which required supporting measures the farmers would need to improve the implementation of the biosecurity measures on their farm?**	**In your opinion, why do you think that these Supporting measures that you have mentioned here (successful and required) are effective to improve biosecurity on the farms?**	**For the required Supporting measures not implemented on the farms that you have mentioned here:**	
				**Could you explain the reasons?**	**What solution or what would it need to implement these supporting measures on the farms?**
Regulations set up supporting biosecurity implementation	□<br>Details<br>………………………………<br>………………………………	□<br>Details<br>…………………………………<br>…………………………………	…………………………………<br>…………………………………<br>…………………………………<br>…………………………………	…………………………………<br>…………………………………<br>…………………………………<br>…………………………………	…………………………………<br>…………………………………<br>…………………………………<br>…………………………………
Other measure 1 in this item<br>………………………<br>……………………..	□<br>Details<br>………………………………<br>………………………………	□<br>Details<br>…………………………………<br>…………………………………	…………………………………<br>…………………………………<br>…………………………………<br>…………………………………	…………………………………<br>…………………………………<br>…………………………………<br>…………………………………	…………………………………<br>…………………………………<br>…………………………………<br>…………………………………
Other measure 2 in this item<br>………………………<br>………………………	□<br>Details<br>………………………………<br>………………………………	□<br>Details<br>…………………………………<br>…………………………………	…………………………………<br>…………………………………<br>…………………………………<br>…………………………………	…………………………………<br>…………………………………<br>…………………………………<br>…………………………………	…………………………………<br>…………………………………<br>…………………………………<br>…………………………………
Other measure 3 in this item<br>………………………<br>………………………	□<br>Details<br>………………………………<br>………………………………	□<br>Details<br>…………………………………<br>…………………………………	…………………………………<br>…………………………………<br>…………………………………<br>…………………………………	…………………………………<br>…………………………………<br>…………………………………<br>…………………………………	…………………………………<br>…………………………………<br>…………………………………<br>…………………………………

Item S6 - Support by a biosecurity advisor (coach /vets)	Which successful supporting measures have already helped the farmers to implement biosecurity measures on their farm?	Which required supporting measures the farmers would need to improve the implementation of the biosecurity measures on their farm?	In your opinion, why do you think that these Supporting measures that you have mentioned here (successful and required) are effective to improve biosecurity on the farms?	For the required Supporting measures not implemented on the farms that you have mentioned here:	
				Could you explain the reasons?	What solution or what would it need to implement these supporting measures on the farms?
Contact support (farm/company visiting)	☐ Details …………………………… ……………………………	☐ Details ……………………………… ………………………………	……………………………… ……………………………… ……………………………… ………………………………	……………………………… ……………………………… ……………………………… ………………………………	……………………………… ……………………………… ……………………………… ………………………………
distance support (by phone, email, Facebook etc.)	☐ Details …………………………… ……………………………	☐ Details ……………………………… ………………………………	……………………………… ……………………………… ……………………………… ………………………………	……………………………… ……………………………… ……………………………… ………………………………	……………………………… ……………………………… ……………………………… ………………………………
advisior coaching methods	☐ Details …………………………… ……………………………	☐ Details ……………………………… ………………………………	……………………………… ……………………………… ……………………………… ………………………………	……………………………… ……………………………… ……………………………… ………………………………	……………………………… ……………………………… ……………………………… ………………………………
Other measure 1 in this item ……………………… ……………………..	☐ Details …………………………… ……………………………	☐ Details ……………………………… ………………………………	……………………………… ……………………………… ……………………………… ………………………………	……………………………… ……………………………… ……………………………… ………………………………	……………………………… ……………………………… ……………………………… ………………………………
Other measure 2 in this item ……………………… ………………………	☐ Details …………………………… ……………………………	☐ Details ……………………………… ………………………………	……………………………… ……………………………… ……………………………… ………………………………	……………………………… ……………………………… ……………………………… ………………………………	……………………………… ……………………………… ……………………………… ………………………………

Other measure 3 in this item<br>………………………<br>………………………	☐<br>Details<br>……………………………….<br>……………………………….	☐<br>Details<br>………………………………….<br>………………………………….	………………………………….<br>………………………………….<br>………………………………….<br>………………………………….	………………………………….<br>………………………………….<br>………………………………….<br>………………………………….	………………………………….<br>………………………………….<br>………………………………….<br>………………………………….
**Item S7 - Organisation of competition for best biosecurity (eg "biosecurity award")**	**Which successful supporting measures have already helped the farmers to implement biosecurity measures on their farm?**	**Which required supporting measures the farmers would need to improve the implementation of the biosecurity measures on their farm?**	**In your opinion, why do you think that these Supporting measures that you have mentioned here (successful and required) are effective to improve biosecurity on the farms?**	**For the required Supporting measures not implemented on the farms that you have mentioned here:**	
				**Could you explain the reasons?**	**What solution or what would it need to implement these supporting measures on the farms?**
Organisation of competition for best biosecurity (eg "biosecurity award")	☐<br>Details<br>……………………………….<br>……………………………….	☐<br>Details<br>………………………………….<br>………………………………….	………………………………….<br>………………………………….<br>………………………………….<br>………………………………….	………………………………….<br>………………………………….<br>………………………………….<br>………………………………….	………………………………….<br>………………………………….<br>………………………………….<br>………………………………….
Other measure 1 in this item<br>………………………<br>………………………	☐<br>Details<br>……………………………….<br>……………………………….	☐<br>Details<br>………………………………….<br>………………………………….	………………………………….<br>………………………………….<br>………………………………….<br>………………………………….	………………………………….<br>………………………………….<br>………………………………….<br>………………………………….	………………………………….<br>………………………………….<br>………………………………….<br>………………………………….
Other measure 2 in this item<br>………………………<br>……………………..	☐<br>Details<br>……………………………….<br>……………………………….	☐<br>Details<br>………………………………….<br>………………………………….	………………………………….<br>………………………………….<br>………………………………….<br>………………………………….	………………………………….<br>………………………………….<br>………………………………….<br>………………………………….	………………………………….<br>………………………………….<br>………………………………….<br>………………………………….
Other measure 3 in this item<br>………………………<br>……………………..	☐<br>Details<br>……………………………….<br>……………………………….	☐<br>Details<br>………………………………….<br>………………………………….	………………………………….<br>………………………………….<br>………………………………….<br>………………………………….	………………………………….<br>………………………………….<br>………………………………….<br>………………………………….	………………………………….<br>………………………………….<br>………………………………….<br>………………………………….

**Item S8 - Financial support for biosecurity implementation**	**Which successful supporting measures have already helped the farmers to implement biosecurity measures on their farm?**	**Which required supporting measures the farmers would need to improve the implementation of the biosecurity measures on their farm?**	**In your opinion, why do you think that these Supporting measures that you have mentioned here (successful and required) are effective to improve biosecurity on the farms?**	**For the required Supporting measures not implemented on the farms that you have mentioned here:**	
				**Could you explain the reasons?**	**What solution or what would it need to implement these supporting measures on the farms?**
Financial support for biosecurity implementation	□<br>Details<br>……………………………<br>……………………………	□<br>Details<br>………………………………<br>………………………………	………………………………<br>………………………………<br>………………………………<br>………………………………	………………………………<br>………………………………<br>………………………………<br>………………………………	………………………………<br>………………………………<br>………………………………<br>………………………………
Other measure 1 in this item<br>………………………<br>………………………	□<br>Details<br>……………………………<br>……………………………	□<br>Details<br>………………………………<br>………………………………	………………………………<br>………………………………<br>………………………………<br>………………………………	………………………………<br>………………………………<br>………………………………<br>………………………………	………………………………<br>………………………………<br>………………………………<br>………………………………
Other measure 2 in this item<br>………………………<br>………………………	□<br>Details<br>……………………………<br>……………………………	□<br>Details<br>………………………………<br>………………………………	………………………………<br>………………………………<br>………………………………<br>………………………………	………………………………<br>………………………………<br>………………………………<br>………………………………	………………………………<br>………………………………<br>………………………………<br>………………………………
Other measure 3 in this item<br>………………………<br>………………………	□<br>Details<br>……………………………<br>……………………………	□<br>Details<br>………………………………<br>………………………………	………………………………<br>………………………………<br>………………………………<br>………………………………	………………………………<br>………………………………<br>………………………………<br>………………………………	………………………………<br>………………………………<br>………………………………<br>………………………………

Item S9 - Other supporting measures (not described above)	Which successful supporting measures have already helped the farmers to implement biosecurity measures on their farm?	Which required supporting measures the farmers would need to improve the implementation of the biosecurity measures on their farm?	In your opinion, why do you think that these Supporting measures that you have mentioned here (successful and required) are effective to improve biosecurity on the farms?	For the required Supporting measures not implemented on the farms that you have mentioned here:	
				Could you explain the reasons?	What solution or what would it need to implement these supporting measures on the farms?
Other measure 1 ……………………… ………………………	☐ Details ……………………………… ………………………………	☐ Details ………………………………… …………………………………	………………………………… ………………………………… ………………………………… …………………………………	………………………………… ………………………………… ………………………………… …………………………………	………………………………… ………………………………… ………………………………… …………………………………
Other measure 2 ……………………… ………………………	☐ Details ……………………………… ………………………………	☐ Details ………………………………… …………………………………	………………………………… ………………………………… ………………………………… …………………………………	………………………………… ………………………………… ………………………………… …………………………………	………………………………… ………………………………… ………………………………… …………………………………
Other measure 3 ……………………… ………………………	☐ Details ……………………………… ………………………………	☐ Details ………………………………… …………………………………	………………………………… ………………………………… ………………………………… …………………………………	………………………………… ………………………………… ………………………………… …………………………………	………………………………… ………………………………… ………………………………… …………………………………
Other measure 4 ……………………… ………………………	☐ Details ……………………………… ………………………………	☐ Details ………………………………… …………………………………	………………………………… ………………………………… ………………………………… …………………………………	………………………………… ………………………………… ………………………………… …………………………………	………………………………… ………………………………… ………………………………… …………………………………

Other measure 5 ………………………. ……………………….	☐ Details ………………………………… …………………………………	☐ Details ………………………………… …………………………………	………………………………… ………………………………… ………………………………… …………………………………	………………………………… ………………………………… ………………………………… …………………………………	………………………………… ………………………………… ………………………………… …………………………………
Other measure 6 ………………………. ……………………….	☐ Details ………………………………… …………………………………	☐ Details ………………………………… …………………………………	………………………………… ………………………………… ………………………………… …………………………………	………………………………… ………………………………… ………………………………… …………………………………	………………………………… ………………………………… ………………………………… …………………………………
